# Influenza‐induced tuft cell expansion is associated with changes in ILC2 populations in the distal lungs of mice

**DOI:** 10.14814/phy2.71000

**Published:** 2026-07-11

**Authors:** Maria Elena Gentile, Michael M. Maiden, Evelyn A. Martinez, Madeline Singh, Harshini Kelam, Nicolas P. Holcomb, Meryl Mendoza, Joanna Wong, Diana M. Abraham, Alena Klochkova, Sara Kass‐Gergi, Andrew E. Vaughan

**Affiliations:** ^1^ Department of Microbiology and Immunology McGill University Montreal Quebec Canada; ^2^ Department of Pulmonary and Sleep Medicine The Children's Hospital of Philadelphia Philadelphia Pennsylvania USA; ^3^ Penn‐CHOP Lung Biology Institute, Perelman School of Medicine University of Pennsylvania Philadelphia Pennsylvania USA; ^4^ Institute for Regenerative Medicine University of Pennsylvania Philadelphia Pennsylvania USA; ^5^ Department of Biomedical Sciences, School of Veterinary Medicine University of Pennsylvania Philadelphia Pennsylvania USA

**Keywords:** ILC2, influenza, lung, tuft cells

## Abstract

Tuft cells act as sentinels that amplify type 2 inflammation primarily by activating type 2 innate lymphoid cells (ILC2s). Although normally absent from the distal lung, ectopic tuft cells form after severe lung injury including influenza infection in mice. Here, we investigated the function of these ectopic tuft cells in shaping innate immunity following influenza injury. We observed that IFNγ deficiency was associated with an increase in tuft cell and ILC2 expansion, whereas ILC2 deficiency was associated with reduced tuft cell expansion, consistent with a reciprocal regulatory axis. Tuft cell–deficient mice exhibited expansion of ILC1s and ILC3s after influenza injury resolution. Single‐cell RNA‐seq of influenza infected whole lung revealed transcriptional signatures consistent with type 1 pathway activation and oxidative stress in the tuft cell‐deficient mice. Following influenza injury and subsequent *Alternaria alternata* challenge, tuft cell–deficient mice also showed neutrophilic and ILC3 expansion. Together, these data are consistent with the presence of a distal‐airway tuft‐cell–ILC2 circuit that may help shape inflammatory responses to viral injury and aeroallergens.

## 1 INTRODUCTION

The distal lung bears appreciable capacity for repair: within gas‐exchanging alveoli, quiescent alveolar type 2 cells can self‐renew and differentiate into alveolar type 1 cells to restore oxygen‐exchanging epithelium following mild injury (Barkauskas et al., 2013; Evans et al., 1975). However, following severe injury such as H1N1 influenza A virus infection, intrapulmonary p63 progenitor cells upregulate cytokeratin 5 (Krt5), migrate, and proliferate into injured alveoli forming “epithelial scars” that restore barrier function at the likely cost of gas exchange (Fernanda de Mello Costa et al., 2020; Vaughan et al., 2015; Xi et al., 2017; Zuo et al., 2015). These dysplastic regions have been found up to 1 year following injury where mice also showed significantly reduced oxyhemoglobin saturations, demonstrating their long‐term impact on lung function (Weiner et al., 2022). Intriguingly, our group and others have identified ectopic tuft cells within these dysplastic regions following influenza A virus (IAV) infection in mice (Barr et al., 2022; Huang et al., 2022; Rane et al., 2019; Roach et al., 2022).

Historically, tuft cells have held varying names depending on anatomic location including brush cells, microvillus cells, and solitary chemosensory cells (Sato, 2007). They are distributed throughout the body including conjunctiva, gingiva, upper airway, thymus, gallbladder, urethra, and intestinal tract (Iqbal et al., 2023). Tuft cells express components of taste transduction signaling pathways, including sweet and umami taste receptors (T1Rs), bitter taste receptors (T2Rs), Gnat3 (α‐gustducin), succinate receptor 1 (SUCNR1), and the transient receptor potential cation channel subfamily M member 5 (TRPM5) to drive chemosensory responses (Lei et al., 2018; Nadjsombati et al., 2018; Strine & Wilen, 2022). Tuft cells also express doublecortin‐like kinase 1 (DCLK1), a protein kinase involved in neuronal migration, a marker commonly used for their identification in situ (Middelhoff et al., 2017). The POU homeodomain transcription factor POU2F3 is necessary for their development (Matsumoto et al., 2011; Yamaguchi et al., 2014; Yamashita et al., 2017). Regardless of their location, tuft cells function as sentinels that detect pathogens—including viruses, bacteria, aeroallergens, and helminths—using taste cell–like chemosensory pathways to orchestrate downstream immune responses (Billipp et al., 2021; Hollenhorst & Krasteva‐Christ, 2023).

CD4^+^ T lymphocytes, known as helper T cells, are classified into Type 1 helper T cells (T_h_1) and Type 2 helper T cells (T_h_2) based on the cytokines they produce (Berger, 2000). T_h_1‐type cytokines are pro‐inflammatory and are responsible for intracellular pathogen killing and autoimmune responses (Berger, 2000). T_h_2‐type cytokines, including interleukins (IL)‐4, IL‐5, and IL‐13, promote atopy through IgE‐mediated eosinophilic responses and anti‐inflammatory effects via IL‐10 (Annunziato et al., 2015; Berger, 2000). At homeostasis, these two arms of the immune system are balanced (Liew, 2002). Innate lymphoid cells (ILCs) act as counterparts to T_h_1/T_h_2 cells, rapidly shaping the early immune environment (Martinez‐Gonzalez et al., 2015). ILC2s promote type 2 immune responses via secretion of type 2 cytokines and are counterbalanced by ILC1s which drive type 1 immune responses via the secretion of IFNγ (Licona‐Limon et al., 2013; Nabekura & Shibuya, 2021). Together, these cells form a reciprocal regulatory axis where the activation of one group of ILCs tilts the overall immune environment toward type 1 or type 2 immunity (von Burg et al., 2015). In parallel, a third immune axis (type 3) driven by ILC3s promotes steroid‐resistant asthma and neutrophilic inflammation through secretion of IL‐17 and IL‐22 (Annunziato et al., 2015).

Tuft cells are well described to form an epithelial‐ILC2 positive feedback circuit in the small intestine and upper respiratory tract. In response to helminth infections in the intestine, tuft cells are the sole producers of IL‐25, which alongside cysteinyl leukotrienes activates ILC2s to produce IL‐13, driving an anti‐parasitic immune response and forming a positive feedback loop that drives further tuft cell expansion through an IL‐4R⍺‐dependent signaling cascade (Bankova et al., 2018; Gerbe et al., 2016; Howitt et al., 2016; von Moltke et al., 2016). Similarly, in patients with chronic rhinosinusitis with nasal polyps, tuft cells are a major source of IL‐25 which drives ILC2 expansion promoting type 2 inflammation and tuft cell differentiation (Kohanski et al., 2018). More recently, rhinovirus infection in immature mice revealed that tuft‐cell–derived IL‐25 induces ILC2 expansion and promotes type 2 inflammation and tuft cell expansion. Importantly, they also demonstrated tuft cell‐dependent airway hyperresponsiveness and mucous metaplasia (Han et al., 2017; Li et al., 2023).

Unexpectedly, given these reports, we previously demonstrated that canonical ILC2‐derived cytokines IL‐4 and IL‐13 have no direct role in ectopic tuft cell differentiation or expansion after H1N1 influenza injury (Barr et al., 2022). Further, we did not observe any obvious effects on epithelial Krt5^+^ dysplasia or goblet cell metaplasia in the absence of tuft cells (Barr et al., 2022). Here, building on our previous findings, we asked whether ectopic tuft cells contribute to an ILC2 response circuit in the distal airways following influenza infection by systematically disrupting key components of the circuit and assessing changes in tuft cell abundance, immune cell populations, and cytokines. We observed that IFNγ deficiency was associated with increased tuft cell and ILC2 expansion, while ILC2 deficiency was associated with reduced tuft cell expansion. We also found that tuft cell‐deficient mice (*Pou2f3* ^−/−^) have reduced eosinophils with concomitant expansion of ILC1s and ILC3s, though proportionally fewer ILC2s, following influenza infection. Further, using single‐cell RNA‐Seq in *Pou2f3* ^−/−^ mice following influenza infection, we found a transcriptomic signature consistent with type 1 pathway activation and oxidative stress. Similarly, in *Pou2f3* ^−/−^ mice first infected with influenza, allowed to recover, and then challenged with the aeroallergen *Alternaria alternata*, we observed an increase in neutrophils and ILC3s. Together, these findings suggest the presence of a distal‐airway tuft cell–ILC2 regulatory circuit that may help shape inflammatory responses in the lower airways to viral infections and aeroallergens.

## 2 RESULTS

### 2.1 IFNγ deficiency is associated with increased tuft cell and ILC2 expansion following influenza infection

**FIGURE 1:**
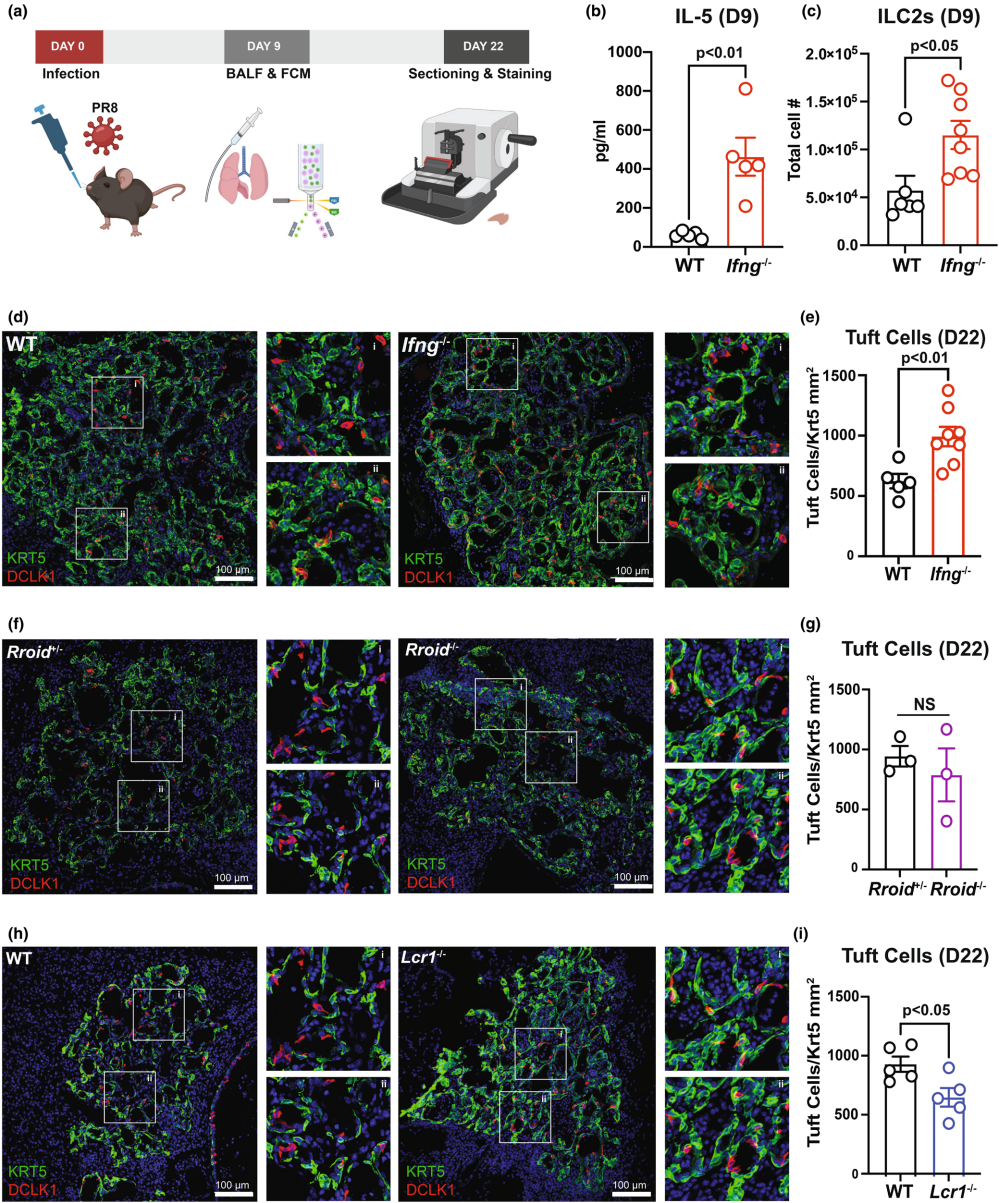
Ectopic pulmonary tuft cells participate in an ILC2‐dependent circuit in the lower airways and are restrained by IFNγ after PR8 infection. (a) Experimental design outlining intranasal PR8 infection followed by collection of bronchoalveolar lavage fluid (BALF) and lungs for flow cytometry (FCM) at day 9 (D9) post‐PR8 infection (p.i.) and immunostaining and tuft cell quantification in the lungs at day 22 (D22) p.i. (b) Protein concentration of IL‐5 in the BALF of *Ifng* ^−/−^mice (*n* = 5) compared to WT C57BL/6J mice (*n* = 5) at D9 p.i. (c) Total cell numbers of type 2 lung innate lymphocytes (ILC2s) (KLRG1^+^NK1.1^−^ of CD127^+^Lineage (Lin)^−^ cells) in *Ifng* ^−/−^mice (*n* = 8) compared to WT C57BL/6J mice (*n* = 6) at D9 p.i. One mouse that lost <15% of starting body weight after influenza infection was excluded from the control group. (d, e) Representative immunostaining and total tuft cell numbers/Krt5+ area in *Ifng* ^−/−^ mice (*n* = 8) compared to WT C57BL/6J controls (*n* = 5), (f, g) in *Rroid* ^−/−^ mice (*n* = 3) compared to *Rroid* ^+/−^ controls (*n* = 3) and in (h, i) in *Lcr1* ^−/−^ mice (*n* = 5) compared to controls (*n* = 5) at D22 p.i. DAPI is stained in blue, Krt5 in green and Dclk1 in red. Insets show magnified views (i, ii). Scale bars are 100 μm and images are cropped from the 20x z‐stack image. (h, i) Two mice that loss <15% of starting body weight after influenza infection were excluded; one in control group and one in *Lcr1* ^−/−^ group. (b, c, e and i) data combined from two‐independent experiments. Each circle represents an individual mouse. *p* values were calculated using unpaired, two‐tailed parametric *t*‐test or Welch's *t*‐test where appropriate to account for non‐normal distribution. The Mann Whitney non‐parametric statistical analyses were also performed where appropriate to confirm findings. (NS = non‐significant). Error bars = SEM.

IFNγ represses allergic inflammation and lower levels of IFNγ are associated with stronger type 2 immune responses (Cautivo et al., 2022; Teixeira et al., 2005). To determine if tuft cell expansion is influenced by this immune context, we infected IFNγ‐deficient mice (*Ifng* ^−/−^) and wild‐type C57BL/6J (WT) mice with influenza (PR8 strain) (Figure 1a). *Ifng* ^−/−^ mice exhibit impaired type 1 immune responses with enhanced susceptibility to intracellular pathogens and exaggerated type 2 inflammation (Dalton et al., 1993).

Accordingly, we observed a significant increase in the type 2 cytokine IL‐5 in BALF (Figure 1b) and a concomitant increase in lung ILC2 cells at day 9 (D9) post PR8‐infection in *Ifng* ^−/−^ versus WT C57BL/6J controls (Figure 1c). These analyses were performed using Welch's *t*‐test, which was selected because of unequal variance between groups. This observed increase in tuft cell numbers may be due to changes in type 2 inflammatory responses at D9 post PR8‐infection.

Changes in body weights were calculated as a proxy for inflammation and disease severity finding no significant difference (Figure S1a). Pulse oximetry was performed prior to infection and at D10, D16, and D21 post PR8‐infection and there was no difference in oxyhemoglobin saturation between *Ifng* ^−/−^ and WT C57BL/6J controls (Figure S1b). Despite this, we detected a significant increase in total tuft cells present within Krt5^+^ areas in *Ifng* ^−/−^ lungs compared to controls 22 days following PR8‐infection (Figure 1d,e), suggesting that unlike Type I and Type III interferons (Barr et al., 2022), IFNγ may contribute to limiting tuft cell expansion through regulation of type 2 inflammatory responses following influenza infection.

Given that IFNγ secretion by ILC1s can constrain type 2 inflammation which could limit tuft cell expansion (Cautivo et al., 2022), we also infected ILC1‐deficient mice (*Rroid* ^*−/−*^) with PR8. *Rroid* ^*−/−*^ mice lack the non‐coding RNA Rroid, which is required for Id2‐dependent development of ILC1s (Mowel et al., 2017). We found ILC1s were dispensable for tuft cell expansion (Figure 1f,g). Again, we measured changes in body weight and performed pulse oximetry, finding no significant differences between *Rroid* ^−/−^ mice and *Rroid* ^+/−^ controls (Figure S1c,d). Together, these data are consistent with a role for IFNγ signaling in constraining tuft cell and ILC2 expansion following influenza infection, similar to its role in regulating the tuft cell–ILC2 circuit in other tissues (Gerbe et al., 2016; Howitt et al., 2016; von Moltke et al., 2016).

### 2.2 ILC2s contribute to the expansion of pulmonary tuft cells following influenza infection

We previously demonstrated that, unlike the gastrointestinal tract, tuft cell expansion post‐influenza is independent of classic ILC2‐derived signals like IL‐4 and IL‐13. To assess whether ILC2s play any remaining role, we infected ILC2‐deficient mice (*Lcr1* ^−/−^) and WT C57BL/6J controls with PR8. *Lcr1* ^−/−^ mice lack an enhancer required to drive *Id2* expression (locus control region 1, *Lcr1*) in ILC2 precursors, blocking their development (Michieletto et al., 2023). Consistent with the literature demonstrating a positive feedback loop between ILC2s and tuft cells (Gerbe et al., 2016; Han et al., 2017; Howitt et al., 2016; Kohanski et al., 2018; Li et al., 2023; von Moltke et al., 2016), we observed a significant reduction in total tuft cell number per Krt5^+^ area in *LCR1* ^−/−^ lungs compared to controls (Figure 1h,i), but no other obvious changes in overt responses to influenza injury (Figure S1e,f). These data suggest that changes in tuft cell number do not impact disease severity or lung damage consistent with our prior findings (Barr et al., 2022), but that lung ILC2s do provide a signal impacting tuft cell differentiation. Together, these data suggest that ectopic tuft cells may participate in an ILC2‐associated circuit in the lower airways.

### 2.3 Tuft cell‐deficiency alters ILC populations following influenza infection

**FIGURE 2:**
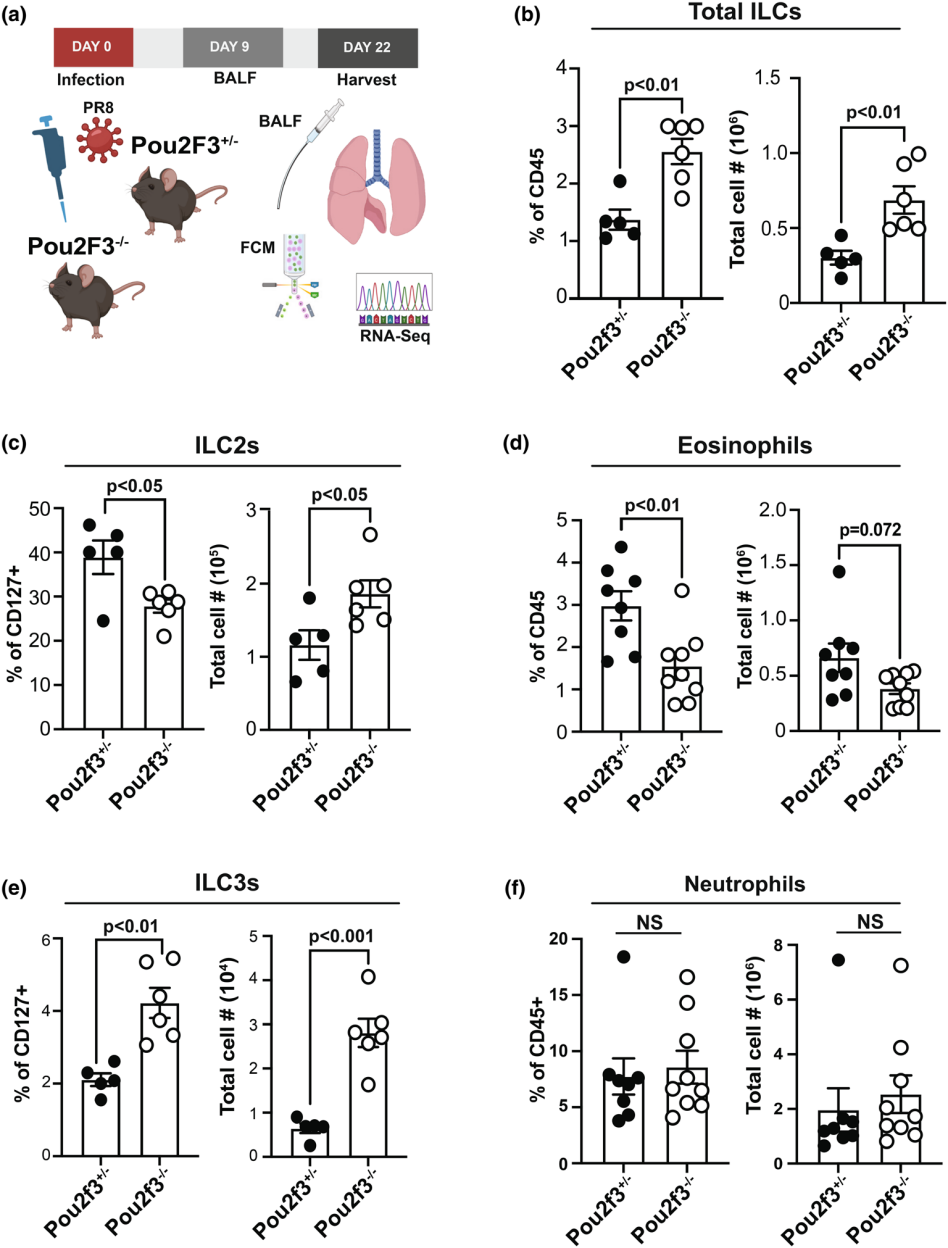
Tuft cell–deficient mice have reduced lung eosinophils and increased ILCs following influenza infection. (a) Experimental design outlining intranasal PR8 infection in *Pou2f3* ^+/−^ and *Pou2f3* ^−/−^ mice followed by collection of bronchoalveolar lavage fluid (BALF) at D9 p.i. and lungs for flow cytometry analysis as well as single‐cell RNA sequencing (RNA‐Seq) at D22 p.i. (b) Frequency of CD45^+^ and total cell numbers of innate lymphocytes (ILCs) (CD127^+^ of Lin^−^CD11b^low/int^ cells) in the lungs of *Pou2f3* ^+/−^ and *Pou2f3* ^−/−^ mice at D22 p.i. (c) Frequency of CD127^+^ and total cell numbers of ILC2s (KLRG1^+^NK1.1^−^ of CD127^+^ Lin^−^ cells) and (d) frequency of CD45^+^ and total cell numbers of eosinophils (CD11c^low/int^ MHCII^−^ of SiglecF^+^F4/80^+^ cells) in the lungs of *Pou2f3* ^+/−^ and *Pou2f3* ^−/−^ mice at D22 p.i. (e) Frequency of CD127^+^ and total cell numbers of ILC3s (Rorgt^+^Tbet^−^ of KLRG1^−^CD127^+^cells) and (f) frequency of CD45^+^ and total cell numbers of neutrophils (Ly6g^+^CD11b^+^ of live CD45^+^ cells) in the lungs of *Pou2f3* ^+/−^ and *Pou2f3* ^−/−^ mice at D22 p.i. (d and f) data combined from two‐independent experiments. Each circle represents an individual mouse. *p* values were calculated using unpaired, two‐tailed parametric Welch's *t*‐test to account for non‐normal distribution. The Mann Whitney non‐parametric statistical analyses were also performed to confirm findings. (NS = non‐significant). Error bars = SEM. Full gating strategy for the different immune cell populations found in material and methods.

Next, we infected global tuft cell‐deficient mice (*Pou2f3* ^−/−^) and heterozygous controls (*Pou2f3* ^+/−^) (Matsumoto et al., 2011) with PR8 and used flow cytometry to examine immune response (Figure 2a). We observed no significant differences in body weight or in total lung cell number in *Pou2f3* ^−/−^ mice compared to controls at D22 post PR8 infection (Figure S2a,b).

When investigating the innate immune cell compartment, we observed an overall increase in ILCs as a percentage of immune cells (CD45^+^) in tuft cell‐deficient mice (Figure 2b). There was also an increase in the absolute number of ILC1 cells compared to controls (Figure S2c). Consistent with the hypothesis that tuft cells drive an ILC2 circuit in the distal airways, there was a reduced relative frequency of ILC2 cells as a percentage of CD127^+^ cells in *Pou2f3* ^−/−^ mice compared to controls (Figure 2c and Figure S2d). However, unexpectedly, the absolute number of ILC2 cells in *Pou2f3* ^−/−^ mice was greater than controls (Figure 2c), seemingly due to expansion of the overall ILC pool (Figure 2b). These analyses were performed using Welch's *t*‐test, which was selected because of unequal variance between groups.

This may partially be explained by ILC2 plasticity following infection. That is, KLRG1 is not exclusively lineage‐defining for ILC2s. However, we observed marked downregulation and instability of the canonical ILC2‐associated transcription factor GATA3 at post‐infection time points, necessitating alternative gating strategies (Roach et al., 2022). This observation is consistent with prior reports demonstrating influenza‐induced loss of GATA3 expression in lung ILC2s accompanied by acquisition of ILC1‐associated markers (Silver et al., 2016). Together, these findings suggest that loss of GATA3 expression may reflect altered lineage identity and functional plasticity within the ILC2 compartment, and the observed reduction in KLRG1^+^ ILC2 frequency may reflect both changes in activation states and abundance.

Further, we observed a significant decrease in eosinophils as a percentage of CD45^+^ cells. However, the reduction in absolute eosinophil number did not reach statistical significance and was influenced by variability within the control group (Figure 2d and Figure S2e). These data are consistent with a trend toward reduced eosinophilic responses.

Finally, we saw an increase in ILC3s (Figure 2e), which are known to drive neutrophil production, recruitment, and activation (Fachi et al., 2024; Ryu et al., 2023), though neutrophil numbers themselves did not differ at this time point (Figure 2f). We also observed no differences in alveolar macrophages, inflammatory monocytes, or natural killer (NK) cells (Figure S2f–h). Together, these data suggest that the presence of tuft cells following viral injury influences some components of type 1 and 3 responses, as reflected by altered ILC composition.

### 2.4 Transcriptional profiling of immune and non‐immune cell types from tuft cell‐deficient mice

Given that we observed limited changes in inflammation and no change in disease severity as measured by weight loss (Figure S2a) after PR8‐infection in *Pou2f3* ^−/−^ mice, we pursued an unbiased approach using single cell RNA sequencing to determine what downstream pathways may be regulated by tuft cells. We profiled both uninfected and post‐influenza mice on D22 following infection, and separated lung cells from these mice into immune (CD45^+^) and non‐immune (CD45^−^) fractions to better resolve the contributions of different cellular populations (Figure 2a).

**FIGURE 3:**
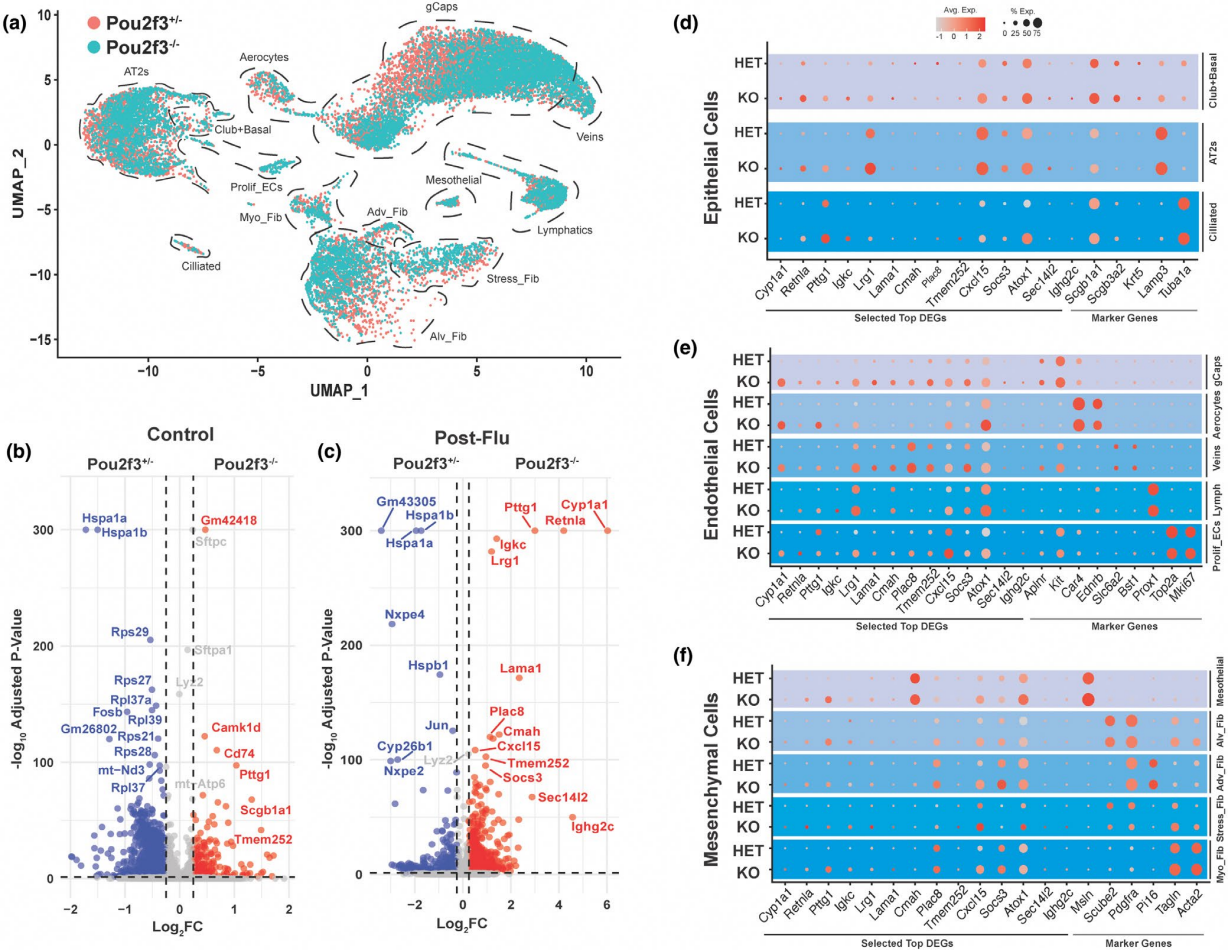
Transcriptional profiling of non‐immune cell populations in tuft cell‐deficient mice post‐influenza. (a) Single‐cell RNA‐seq UMAP clustering of sorted non‐immune (CD45^−^) cell types from mice at D22 p.i. (b) Volcano plot comparing gene expression in uninfected *Pou2f3* ^+/−^ and *Pou2f3* ^−/−^ mice. (c) Volcano plot comparing gene expression in infected *Pou2f3* ^+/−^ and *Pou2f3* ^−/−^ mice. Top 14 differentially expressed genes (DEGs) and selected marker genes in (d) epithelial, (e) endothelial, and (f) mesenchymal cells from *Pou2f3* ^+/−^ and *Pou2f3* ^−/−^ mice at D22 p.i.

In non‐immune cell types, UMAP visualization revealed global differences in gene expression between *Pou2f3* ^−/−^ mice and controls with transcriptional differences most apparent in general capillaries (gCaps) and alveolar fibroblast (Alv_Fib) (Figure 3a). At D22 following PR8 influenza infection, 848 genes were differentially expressed in *Pou2f3* ^−/−^ mice compared with control animals (adjusted *p* <0.05) (Figure 3b,c). There were a total of 13 genes upregulated and 14 genes downregulated in *Pou2f3* ^−/−^ mice regardless of infection status. The top 20 differentially expressed genes (DEG) for epithelial, endothelial, and mesenchymal along with marker genes cells are shown in Figure 3d–f. The transcriptomic profile was consistent with type 1 immune pathway activation, including differential expression of *Retnla*, which suppresses type 2 immunity in epithelial cells (Pesce et al., 2009); *Cxcl15*, a neutrophil‐recruiting chemokine expressed in mesenchymal cells; and *Cyp1a1*, which mitigates oxidative stress (Lingappan et al., 2014). *Atox1* was also differentially expressed across all cell types and is known to prevent oxidative damage (Hatori & Lutsenko, 2016). We also saw differential expression of *Socs3*, which shifts macrophages toward pro‐inflammatory phenotypes and limits IL‐10 anti‐inflammatory signaling (Berlato et al., 2002).

Overall, immune cell types exhibited fewer transcriptomic differences compared to non‐immune cells (Figure S3). We observed differential expression of 556 genes between *Pou2f3* ^−/−^ and controls at D22 post PR8‐infection (adjusted *p* <0.05) (Figure S3b,c). In immune cell types, there were 9 genes upregulated and 15 genes downregulated in *Pou2f3* ^−/−^ mice, independent of infection status. The observed differential expression was primarily restricted to mitochondrial and housekeeping genes which may indicate apoptosis or changes in metabolic states. (e.g., *mt‐Co3, mt‐Atp6, mt‐Co2, mt‐Cytb, mt‐Nd* genes, *Ubc, Rps29*) (Figure S3d,e). Notably, *Pou2f3* ^−/−^ cells showed consistently higher mitochondrial gene expression, particularly in T and NK cells, suggesting that the *Pou2f3* genotype may alter cellular metabolic or activation states without affecting lymphocyte identity.

To further explore differences in inflammatory response as a function of tuft cells, we also performed a Luminex® cytokine array on BALF collected from *Pou2f3* ^−/−^ mice and controls at D9 and D22 post PR8‐infection (Figure 2a and Figure S4). Though not significant, we note a trending increase in the neutrophil chemoattractant CXCL5/LIX (Figure S4k) in *Pou2f3* ^−/−^ mice only, consistent with prior reports that tuft cell‐deficient animals have increased CXCL5/LIX expression and neutrophilia in the gall bladder (O'Leary et al., 2022). We found no statistically significant differences in cytokine profiling in *Pou2f3* ^−/−^ mice compared to controls (Figure S4a–m).

Taken together, these findings suggest an unanticipated role for tuft cells in influencing type 1‐ and type 3‐associated responses, including the potential to recruit neutrophils and modulate ILC expansion during recovery from influenza injury.

### 2.5 Tuft cell‐deficient mice have an increased pulmonary neutrophilic response following *Alternaria* challenge

**FIGURE 4:**
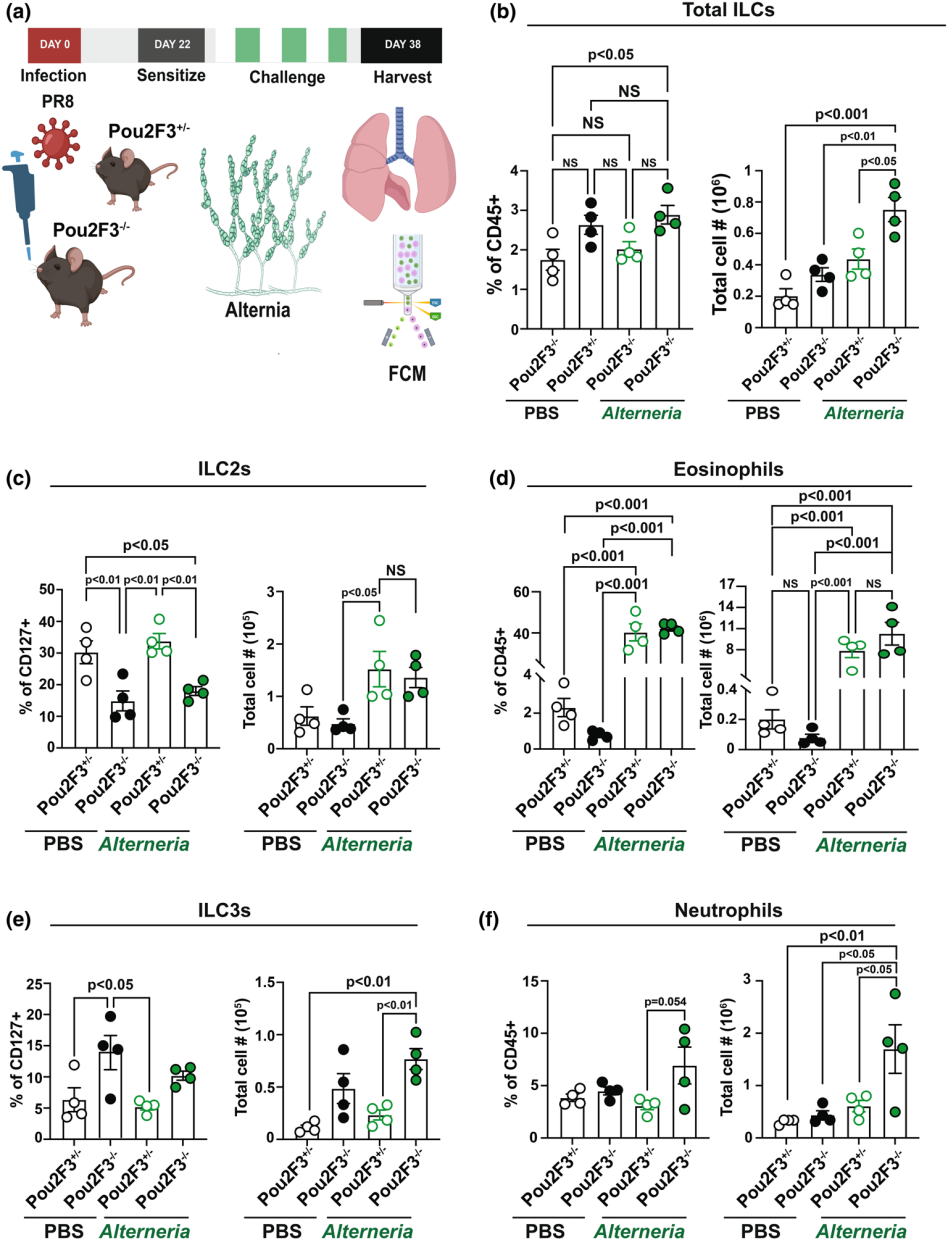
Pulmonary neutrophil and ILC responses are elevated in tuft cell–deficient mice after *Alternaria alternata* secondary challenge. (a) Experimental design outlining intranasal PR8 infection followed by allergen sensitization (40 μg) and challenge (20 μg) as well as collection of lungs for flow cytometry analysis in *Pou2f3* ^+/−^ and *Pou2f3* ^−/−^ mice at D38 p.i. (b) Frequency of CD45^+^ and total cell numbers of ILCs (CD127^+^ of Lin^−^CD11b^low/int^ cells) in *Alternaria alternata* or PBS challenged *Pou2f3* ^+/−^ and *Pou2f3* ^−/−^ mice at D38 p.i. (c) Frequency of CD127^+^ cells and total cell numbers of ILC2s (KLRG1^+^NK1.1^−^ of CD127^+^ Lin^−^ cells) and (d) frequency of CD45^+^ cells and total cell numbers of eosinophils (CD11c^low/int^ MHCII^−^ of SiglecF^+^CD11B^int/high^ cells) in the lungs of *A. alternata* or PBS challenged *Pou2f3* ^+/−^ and *Pou2f3* ^−/−^ mice at D38 p.i. (e) Frequency of CD127^+^ cells and total cell numbers of ILC3s (Rorgt^+^ of KLRG1^−^NK1.1^−^CD127^+^ cells) and (f) frequency of CD45^+^ cells and total cell numbers of neutrophils (Ly6g^+^CD11b^+^ of live CD45^+^ cells) in the lungs of *A. alternata* or PBS challenged *Pou2f3* ^+/−^ and *Pou2f3* ^−/−^ mice at D38 p.i. Each circle represents an individual mouse. *p* values were calculated using one‐way ANOVA with Tukey's post‐test for multiple comparisons (NS = non‐significant). Error bars = SEM. Complete gating strategy for the different immune cell populations found in material and methods.

As previously noted, *Pou2f3* ^−/−^ mice infected with PR8 had a decreased frequency of ILC2s and eosinophils (Figure 2c,d). Given their established role in type 2 inflammation in the upper airways (Han et al., 2017; Iqbal et al., 2023; Kohanski et al., 2018; Li et al., 2023), we hypothesized that tuft cells may promote a pro‐asthmatic immune milieu in the distal airways following influenza infection. To test this, we used a two‐hit model in which mice were first infected with PR8, allowed to recover for 3 weeks, and then challenged with the aeroallergen *Alternaria alternata* followed by flow cytometry to assess the inflammatory contribution of tuft cells (Gorska, 2018) (Figure 4a).

We observed no change in body weight throughout the course of the experiment (Figure S5a). However, we did observe an increase in total cell numbers in the lung upon *A. alternata* challenge regardless of genotype (Figure S5b). At day 37 following PR8 infection, pulse oximetry measurements revealed a significant decrease in oxyhemoglobin concentrations in *Pou2f3* ^+/−^ control mice exposed to *A. alternata*, consistent with impaired gas exchange (Figure S5c). In contrast, *Pou2f3* ^−/−^mice showed no significant change in oxyhemoglobin saturations. These data suggest that tuft cells may play a role in suppressing airway inflammation following aeroallergen challenge.

Next, we assessed the impact of *Pou2f3* deletion on innate immune cell populations. We observed a significant increase in total ILCs in *Pou2f3* ^−/−^mice challenged with *A. alternata* compared to controls (Figure 4b). There was also an increase in the absolute number of ILC1s in mice challenged with *A. alternata* relative to PBS challenge, regardless of genotype (Figure S6a). In these experiments, ILC1s were identified as KLRG1^−^NK1.1^+^ cells due to fluorochrome limitations. This gating strategy provides less phenotypic specificity than KLRG1^−^RORγt^−^T‐bet^+^ gating as previously mentioned (Silver et al., 2016) and may capture a broader subset of ILC1s. In keeping with a role for tuft cells in an epithelial‐ILC2 circuit, *Pou2f3* ^−/−^ mice had decreased frequencies of pulmonary ILC2s relative to Pou2f3^+/−^ controls (Figure 4c). There was no significant change in total cell numbers (Figure 4c), likely due to expansion of the overall ILC pool (Figure 4b). Surprisingly, there was no difference in pulmonary eosinophils between *Pou2f3* ^−/−^ and *Pou2f3* ^+/−^ mice challenged with *A. alternata* at D38 following PR8 infection (Figure 4d).

Analysis of ILC3 populations, which drive neutrophilic inflammation (Fachi et al., 2024; Ryu et al., 2023), following *A. alternata* challenge revealed significantly increased total numbers in *Pou2f3* ^−/−^ mice compared to *Pou2f3* ^+/−^ controls (Figure 4e). Intriguingly, there was a significant increase in lung neutrophils in *Pou2f3* ^−/−^ mice challenged with *A. alternata* compared to *Pou2f3* ^+/−^ mice (Figure 4f). We also found no differences in additional immune cell types including alveolar macrophages, inflammatory monocytes, or NK cells between *Pou2f3* ^−/−^ and *Pou2f3* ^+/−^ mice challenged with *A. alternata* (Figure S6b–d). Taken together, these data suggest that tuft cells partially constrain neutrophilic inflammation following aeroallergen challenge.

## 3 DISCUSSION

Although tuft cells are critical chemosensory regulators of immunity to helminths and bacteria, their responses to viral infections and aeroallergens remain poorly understood, especially in the context of the distal airways (Iqbal et al., 2023). In the upper respiratory tract and intestine, tuft cells regulate an epithelial‐ILC2 circuit that drives type 2 immunity that is dependent on both IL‐25 and IL‐4r⍺ signaling (Gerbe et al., 2016; Howitt et al., 2016; von Moltke et al., 2016). Conversely, our previous work demonstrated that post‐IAV ectopic tuft cells develop independently of IL‐25 and IL‐4r⍺ signaling (Barr et al., 2022) but still rely upon the master transcription factor POU2F3 for their development (Barr et al., 2022) and share transcriptomic features with both intestinal and tracheal tuft cells, suggesting analogous functional roles (Hollenhorst & Krasteva‐Christ, 2023). To explore these possible functions as well as what upstream signals are required for tuft cell development, we genetically disrupted key components of the ILC2‐circuit and assessed their relative contributions to circuit function.

Our findings generally reinforce a role for tuft cells within an epithelial–ILC2 regulatory circuit in the lower airways, albeit with some interesting differences compared to existing models. IFNγ can suppress ILC2 cytokine production (Duerr et al., 2016; von Burg et al., 2015), and accordingly, we found that IFNγ deficiency was associated with ILC2 and tuft cell expansion (Figure 1c–e). In contrast, ILC2 deficiency was associated with reduced post‐influenza tuft cell expansion (Figure 1h,i), suggesting a reciprocal regulatory axis between tuft cells and ILC2s. While we presume the effects of IFNγ are acting largely through inhibition of ILC2 expansion (Cautivo et al., 2022), we cannot exclude the broader effects mediated through the suppression of type 2 inflammation nor direct suppressive effects of IFNγ on the basal cell progenitors themselves. Additional studies will be required to distinguish between these possibilities. Further, the identity of the ILC2‐derived signal promoting tuft cell differentiation remains unknown, an especially intriguing open question given previous data suggesting neither IL‐4 nor IL‐13 are critical for their development post‐influenza.

We did not observe an obviously attenuated injury or inflammatory response in tuft cell‐deficient mice following influenza infection, likely due to the emergence of ectopic tuft cells in the distal airways during the recovery phase. Upon recovery from influenza infection, tuft cell–deficient mice exhibited a reduction in frequency of lung eosinophils and ILC2s (Figure 2c,d). We also observed an expansion of ILC1s and ILC3s (Figure S2c and Figure 2e) as well as a trend toward increased CXCL5/LIX in BALF (Figure S4k) at D22 post PR8‐infection. Given the relatively small sample sizes and non‐normal distribution of several analytes as many samples were below the limit of detection, cytokine profiling results should be interpreted cautiously.

In keeping with these observations, a variety of gene expression changes observed in single‐cell RNA‐seq further suggested activation, or at least poised activation, of type 1 inflammation‐associated pathways in tuft cell–deficient mice following influenza infection. Therefore, we challenged *Pou2f3* ^−/−^ mice with the fungus *A. alternata* to see if they would have a decreased asthmatic phenotype. We did not observe a significant decrease in eosinophil or ILC2 numbers in tuft cell‐deficient mice challenged with *A. alternata* compared to controls (Figure 4c,d). Instead, we observed an increase in neutrophils and ILC3 numbers (Figure 4e,f).

Though the lack of tuft cell‐dependent changes in eosinophilia was somewhat surprising, we predict that the prolonged sensitization to *A. alternata* extract over several weeks may result in antigen‐specific T_h_2 T cell responses overriding the influences of more acute innate type 2 responses promoted by tuft cells and ILC2s. Therefore, this aeroallergen protocol is not well suited to distinguish the innate tuft cell–ILC2 circuit from adaptive Th2 responses in the distal airways, which is an important limitation of the current study and an area for future investigation using more acute allergen exposure models. An additional limitation of this model is that tuft cell‐deficient mice lack tuft cells globally. Therefore, we cannot definitively exclude contributions from resident tuft cell populations in the upper airways or trachea or distinguish whether the observed immunologic changes are specific to the post‐influenza context versus a broader consequence of tuft cell deficiency during allergen challenge. Further, baseline transcriptional differences were observed between uninfected control and tuft cell‐deficient lungs, suggesting broader genotype‐associated effects independent of injury or aeroallergen challenge.

Another limitation of our analysis is that inflammatory conditions during influenza recovery may promote ILC plasticity, including conversion of ILC2s toward ILC1‐like phenotypes characterized by loss of canonical ILC2 markers as described by Silver et al. (Silver et al., 2016). Therefore, the observed increase in ILC1s and reduction in ILC2s in tuft cell‐deficient mice may reflect phenotypic conversion rather than changes in lineage distributions. Nevertheless, our findings are suggestive of a role for ectopic tuft cells in shaping ILC dynamics and inflammatory responses following viral injury.

Previously, we found that tuft cells are not required for the formation of Krt5^+^ cells, goblet cells, or conversion of Krt5^+^ cells to AT2s after severe influenza injury (Barr et al., 2022), though this does not preclude the possibility of tuft cells contributing to differential inflammatory responses or other less obvious effects. For example, although our prior work demonstrated that IL‐25 and IL‐4ra signaling appears dispensable for influenza‐induced ectopic tuft cell development in the distal lungs, this does not exclude the possibility that IL‐25 may still contribute to downstream tuft cell–ILC2 communication after tuft cells are established. Further, because the LCR1‐deficient mice lack ILC2s developmentally, we cannot exclude compensatory immune changes in other ILC2‐associated mediators such as amphiregulin, IL‐9, or cysteinyl leukotrienes that may contribute to tuft cell expansion in the distal lung (Shang et al., 2025).

While our findings are consistent with a role for tuft cell–ILC interactions in shaping inflammatory responses following influenza injury, additional studies using tissue‐specific inducible tuft cell knockout models that allow us to specifically and temporally ablate ectopic tuft cells after they've developed will be required to establish causality more definitively. Taken together, these findings highlight unexpected differences and complexities in tuft cell signaling in the lung compared to more well‐studied tissues like the intestines.

## 4 METHODS

### 4.1 Animals

Mice were housed under specific pathogen‐free conditions in ventilated cages with controlled temperature and humidity on a 12‐h light/dark cycle and were provided food and water ad libitum. Animals were fed a standard chow diet (PicoLab Rodent Diet 20 Cat#: 5053). C57BL6/J (RRID:IMSR_JAX:000664), *Ifng* ^−/−^ (RRID:IMSR_JAX:008228) (Dalton et al., 1993), *Rroid* ^+/−^, *Rroid* ^−/−^ (Mowel et al., 2017), *LCR1* ^−/−^ (Michieletto et al., 2023), *Pou2f3* ^+/−^, and *Pou2f3* ^−/−^ (Matsumoto et al., 2011) mice were used. Adult mice of both sexes were used, and all mice are on a C57BL6/J background unless otherwise noted. All animal procedures were approved by the University of Pennsylvania Institutional Animal Care and Use Committee (IACUC; protocol 806262). At experimental endpoints mice were euthanized by inhaled isoflurane overdose in an induction chamber followed by exsanguination via transection of the abdomen to ensure death, in accordance with IACUC guidelines. For all animal studies, no statistical method was used to predetermine sample size. The experiments were not randomized, and the investigators were not blinded to allocation during experiments and outcome assessment.

### 4.2 IAV infection and aeroallergen challenge

Infections were performed as previously described (Barr et al., 2022). All viral infections utilized influenza strain A/H1N1/PR/8 obtained from Dr. Carolina Lopez (Garcia et al., 2020). For influenza infection at the University of Pennsylvania, virus was administered intranasally. Mice ranging between 15 and 20 g in weight were infected with 30 tissue culture infectious dose (TCID) 50 units of PR8, mice weighing between 20 and 25 g were given 40 TCID50 units, and mice ranging between 25 and 30 g in weight were given 50 TCID50 units. For aeroallergen experiments, mice were first sensitized with 40 μg of *Alternaria alternata* extract intranasally on D22 post PR8‐infection. Mice were then challenged with 6 doses of *A. alternata* intranasally (20 μg) to induce allergen sensitivity on days D29, D30, D31, D33, D34, and D35 post‐PR8 infection as previously described (Gorska, 2018; Michieletto et al., 2023). Control mice received Phosphate‐Buffered Saline (PBS). PR8‐infected mice that did not survive to the end point of the experiment or lost less than 15% of starting body weight were excluded from analysis.

### 4.3 Whole‐lung single‐cell suspension preparations

Mice were euthanized by inhaled isoflurane overdose in an induction chamber followed by exsanguination via transection of the abdominal to ensure death, in accordance with IACUC guidelines. Lungs were then harvested from mice and single‐cell suspensions were prepared. Briefly, lungs were perfused with ice cold PBS via the left atrium, lobes were separated and collected in ice cold Dulbecco's Modified Eagle Medium (DMEM; Thermo Fisher Scientific) +10% fetal bovine serum (FBS; Thermo Fisher Scientific). Once all mice were harvested, the lobes were placed in 1.5 mL of digestive media (DMEM with 0.15 mg/mL of Liberase TM (Roche # 5401119001)), 0.014 mg/mL of DNase I (Sigma Aldrich # D4527‐20KU) and 0.20 mg/mL of Dispase II (Sigma Aldrich D4693‐1G), cut into small pieces and placed at 37°C to shake for 40 min. The samples were then passed through a 16‐G needle three times and placed back shaking at 37°C for 15 min. The samples were then passed through an 18‐G needle three times and filtered through a 70 μm strainer. The filters were washed with DMEM+10% FBS. Red blood cells were then lysed for 3 min (Thermo Fisher Scientific #A1049201) and cells were manually counted using a hemacytometer. Whole‐lung single‐cell suspensions were then used for subsequent experiments.

### 4.4 Flow cytometry for immunophenotyping

Cell suspensions were washed twice in PBS and then incubated with a fixable viability dye (eFluor 780 eBioscience # 65‐0865‐18) for 20 mins at 4°C. Afterwards, the cells were incubated with TruStain FcX (anti‐mouse CD16/32) Antibody (BioLegend, #101319) for 10 mins at 4°C. The cells were then stained with the following antibodies in appropriate combinations of fluorophores. B220 (clone: RA3‐6B2), CD3 (clone: 145‐2C11), CD11b (clone: M1/70), CD11c (clone: N418), CD45 (clone: 30‐F11), CD127 (clone: A7R34), F4/80 (clone: BM8), KLRG1 (clone: 2F1/KLRG1), Ly6c (clone: HK1.4), Ly6g (clone: 1A8), MHCII (clone: M5/114.15.2), NK1.1 (clone: PK136), Rorgt (clone: Q31‐378), Siglec‐F (clone: E50‐2440), and Tbet (clone: 4B10). The samples were then fixed with 3.2% paraformaldehyde for 12 min at 4°C. For intracellular staining of transcription factors, cells were fixed and permeabilized with the FoxP3 Fix/Perm kit (eBiosciences; Cat# 00‐5523‐00) according to the manufacturer's instructions. Data were acquired with a FACSymphony A3 (BD Biosciences) and analyzed using FlowJo software.

Gating strategy for Figure 1c, Figure 2 and Figure S2 defining: Alveolar Macrophages (singlet Live CD45^+^Ly6g^−^ Ly6c^low/int^ F4/80^+^SiglecF^+^MHCII^+^CD11c^+^), Eosinophils (singlet Live CD45^+^Ly6g^−^ Ly6c^low/int^ F4/80^+^SiglecF^+^MHCII^−^CD11c^low/int^), ILCs (singlet Live CD45^+^Lin^−^ (CD3^−^B220^−^) CD11b^int/low^CD127^+^), ILC1s (singlet Live CD45^+^Lin^−^CD11b^int/low^CD127^+^KLRG1^−^Rorgt^−^Tbet^+^). Consistent with prior reports of influenza‐induced ILC2 plasticity and reduced GATA3 expression (Silver et al., 2016), KLRG1 was used as a surface marker of activated ILC2s. ILC2s (singlet Live CD45^+^Lin^−^CD11b^int/low^CD127^+^NK1.1^−^KLRG1^+^), ILC3s (singlet Live CD45^+^Lin^−^CD11b^int/low^CD127^+^KLRG1^−^ Tbet^−^ Rorgt^+^), Inflammatory Monocytes (singlet Live CD45^+^Ly6g^−^CD11b^+^Ly6c^high^), Natural Killer cells (singlet Live CD45^+^Lin^−^ CD11b^int/low^CD127^−^NK1.1^+^) and Neutrophils (singlet Live CD45^+^CD11b^+^Ly6g^+^).

Gating strategy for Figure 4 and Figure S6 defining Alveolar Macrophages (singlet Live CD45^+^Ly6g^−^SiglecF^+^CD11b^int/high^MHCII^+^CD11c^+^), Eosinophils (singlet Live CD45^+^Ly6g^−^SiglecF^+^CD11b^int/high^ MHCII^−^CD11c^low/int^). NK1.1 expression was used as a surface marker surrogate for T‐bet due to fluorochrome limitations within the staining panel. ILCs (singlet Live CD45^+^Lin^−^ (CD3^−^B220^−^) CD11b^int/low^CD127^+^), ILC1s (singlet Live CD45^+^Lin^−^CD11b^int/low^CD127^+^KLRG1^−^NK1.1^+^), ILC2s (singlet Live CD45^+^Lin^−^CD11b^int/low^CD127^+^NK1.1^−^KLRG1^+^), ILC3s (singlet Live CD45^+^Lin^−^CD11b^int/low^CD127^+^KLRG1^−^ NK1.1^−^ Rorgt^+^), Inflammatory Monocytes (singlet Live CD45^+^Ly6g^−^SiglecF^−^ CD11b^+^Ly6c^high^), Natural Killer cells (singlet Live CD45^+^Lin^−^ CD11b^int/low^CD127^−^NK1.1^+^), and Neutrophils (singlet Live CD45^+^CD11b^+^Ly6g^+^).

### 4.5 Fluorescence‐activated cell sorting for single cell RNA‐Seq

Whole‐lung single‐cell suspensions were prepared as above and as previously described (Barr et al., 2022). First, whole‐lung single‐cell suspensions were pelleted for 5‐mins at 550×g at 4°C. Pellets were re‐suspended in sort buffer (SB; DMEM+2% Cosmic Calf serum (CC; Thermo Fisher Scientific; Cat#: #: SH30087.03) + 1% penicillin and streptomycin). Cells were blocked with 1:50 TruStain FcX (anti‐mouse CD16/32 antibody; BioLegend, #101319) for 10‐mins at 37°C. For immune cells we performed EasyStep™ Mouse CD45 Positive Selection Kit (StemCell Technologies; Cat#: 18945) per manufacture's protocol. For nonimmune cells, the cell suspension was stained using allophycocyanin/Cy7 conjugated rat anti‐mouse CD45 antibody (1:200, BioLegend, #101319), PE‐conjugated rat anti‐mouse EpCam antibody (1:500, BioLegend, G8.8, #118206). Stained cells and ‘fluorescence minus one’ controls were then resuspended in SB + 1:1000 Dnase +1:1000 Draq7 (BioLegend, #424001) as a live/dead stain. Nonimmune cells were isolated as all live CD45^neg^ cells. All FACS sorting was done on a BD FACS AriaFUSION Sorter (BD Biosciences).

### 4.6 Single‐cell RNA‐seq

Whole‐lung single‐cell suspensions were prepared as above and as previously described (Barr et al., 2022). Single‐cell RNA‐Seq was performed using the Chromium System (10× Genomics) and the Chromium Single Cell 3’ Reagent Kits v2 (10× Genomics) at the Children's Hospital of Philadelphia Center for Applied Genomics. After sequencing, initial data processing was performed using Cellranger (v.3.1.0). Cellranger mkfastq was used to generate demultiplexed FASTQ files from the raw sequencing data. Next, Cellranger count was used to align sequencing reads to the mouse reference genome (GRCm38) and generate single‐cell gene barcode matrices. Post‐processing and secondary analysis were performed using the Seurat package (v.4.4.1). First, variable features across single cells in the dataset will be identified by mean expression and dispersion. Identified variable features were then used to perform a principal component analysis (PCA). The dimensionally reduced data was used to cluster cells and visualize using a Uniform Manifold Approximation and Projection (UMAP) plot.

### 4.7 Tissue preparation for immunofluorescence

Each lung was prepared as previously described (Barr et al., 2022). Briefly, lungs were perfused with PBS via the left atrium and then perfused with ice‐cold 3.2% paraformaldehyde (PFA; Thermo Fisher Scientific) and placed in a 50 mL tube with 25 mL of PFA to shake at room temperature (RT) for 1 h. Following incubation, the lungs were washed twice in PBS for 1 h at RT. Lungs were then placed in 30% sucrose (Sigma‐Aldrich) overnight shaking at 4°C. The following day, the tissues were placed in 15% sucrose‐50% optimal cutting temperature compound (OCT; Fisher Healthcare) shaking for 2 h at RT. The fixed lungs were then embedded in OCT and flash frozen. Using a cryostat, the lungs were sectioned (6 μm) and stored at −20°C. Lung sections were fixed in 4% PFA for 5 mins at RT and then washed three times with PBS for 5 mins at RT. Slides were blocked for 1 h at RT in a humid chamber with blocking buffer: 1% BSA, Gold Bio, 5% donkey serum (Sigma; Cat#: D9663), 0.1% Triton X‐100 (Fisher BioRe‐agents) and 0.02% sodium azide (Sigma‐Aldrich) in PBS. The slides were then stained in blocking buffer overnight at 4°C with a combination of primary antibodies. The following day, the slides were rinsed 3 times for 2 mins with PBS + 0.1% Tween (Sigma‐Aldrich) and then stained with secondary antibodies in blocking buffer for 90 mins at RT in the humidity chamber. The slides were rinsed 3 times for 2 mins with PBS + 0.1% Tween and stained with DAPI (1:10,000 dilution; Cat#: D21490, Thermo Fisher Scientific). Slides were then mounted with Fluoroshield (Sigma) and imaged using a Leica inverted fluorescent microscope Dmi8 and analyzed using Las X software. Primary antibodies used: rabbit anti‐Dclk1 (1:500 dilution; Cat#: ab37994 or ab31704, Abcam), chicken anti‐Krt5 (1:1000 dilution; Cat#: 905901, BioLegend). Secondary antibodies used: donkey anti‐rabbit AF568 (1:1000 dilution; Cat#: A10042, Thermo Fisher Scientific), donkey anti‐chicken AF488 (1:1000 dilution; Cat#: 703‐545‐155, Jackson ImmunoResearch).

### 4.8 Pulse oximetry

Peripheral oxygen saturation (SpO2) was determined as previously described (Weiner et al., 2022). Briefly, using a MouseOx Plus Rat & Mouse Pulse Oximeter and a MouseOx small collar sensor (Starr Life Sciences Corp.), SpO2 was taken at the indicated time points for each experiment. At least 1 day prior to the initial reading, the mouse's neck and shoulder area was shaved with a razor at the site where the collar sensor was placed. Recordings were taken using MouseOx Premium Software (Starr Life Sciences Corp., Oakmont, PA, USA). Only measurements with zero error codes were used in our analysis. The overall average of the readings was used to determine the SpO2 for each mouse at the indicated time points.

### 4.9 Image quantification

Lung sections were imaged on a Leica Dmi8 microscope using a 20 × 0.75 NA objective. Image quantification was performed by manual area measurements utilizing ImageJ/FIJI for Krt5 area and manual quantification of Dclk1+ cells. We counted the total tuft cell number in ≥ 2 discrete Krt5^+^ cell areas in total lung area by scanning ≥ 3 whole lobe sections/sample to ensure adequate representation.

### 4.10 Chemokine/cytokine screen

Cytokines and chemokines were measured using the MILLIPLEX® MAP Mouse Cytokine/Chemokine Magnetic Bead Panel – Premixed 32 Plex Immunology Multiplex Assay (MilliporeSigma; Cat#: MCYTMAG‐70 K‐PX32) based on Luminex® xMAP® technology (Lombardelli et al., 2021). Briefly, bronchoalveolar lavage fluid (BALF) was collected from mice using ice‐cold PBS (1 mL) as previously described (Xi et al., 2017). Samples were incubated with antibody‐conjugated fluorescent microspheres, followed by biotinylated detection antibodies and streptavidin‐phycoerythrin. Beads were analyzed on a Luminex® platform, and analyte concentrations were calculated from standard curves using five‐parameter logistic regression performed by the Human Immunology Core at the University of Pennsylvania. For analysis, values below the assay limit of detection were assigned the lower limit of detection for visualization and statistical analysis.

### 4.11 Statistics

All statistical calculations were performed using GraphPad Prism. Relevant statistical tests utilized are stated in the figure legends. For comparisons between two groups, Welch's two‐tailed *t*‐tests were used to account for unequal variance between groups where appropriate. Although several datasets demonstrated biological variability and modest sample sizes, Welch's testing was selected because of its relative robustness to unequal variance and moderate deviations from normality. Nonparametric Mann–Whitney analyses were also performed where appropriate to confirm major findings, particularly for cytokine array data given small sample sizes.

## AUTHOR CONTRIBUTIONS

Michael M. Maiden, Maria Elena Gentile, Conceptualization, Data curation, Formal analysis, Investigation, Methodology, Writing – original draft, Writing – review and editing; Evelyn A. Martinez, Data curation; Madeline Singh, Data curation; Harshini Kelam, Data curation; Alena Klochkova, Sara Kass‐Gergi, Joana Wong, Nicolas P. Holcomb, Meryl Mendoza and Diana M. Abraham, Writing – review and editing; Andrew E. Vaughan, Conceptualization, Resources, Data curation, Formal analysis, Supervision, Funding acquisition, Investigation, Methodology, Writing – original draft, Project administration, Writing – review and editing.

## FUNDING INFORMATION

This work was supported by National Institutes of Health (T32HL160493—Lisa Young, Sharon McGrath), (RO1HL153539—Andrew E Vaughan).

## ETHICS STATEMENT

All animal procedures were approved by the Institutional Animal Care and Use Committee (IACUC; protocol 806262) of the University of Pennsylvania. All experiments were performed with every effort to minimize suffering.

## Supporting information


**Figure S1.** Changes in body weight and oxyhemoglobin saturations following PR8 infection. Body weight and pulse oximetry were assessed prior to infection and at indicated time points following PR8 infection. (A, B) WT C57BL/6J (*n* = 2–5) and *Ifng* ^−/−^ (*n* = 3–8) mice, (C, D) *Rroid* ^+/−^ (*n* = 3), and *Rroid* ^−/−^ (*n* = 3) mice and (E, F) *Lcr1* ^‐/−^ (*n* = 3–5) and WT C57BL/6J (*n* = 3–5) mice. (A, E) combined two independent experiments. (B, D and F) each circle represents an individual mouse. (E) Two mice that lost <15% of starting body weight after influenza infection were excluded, one from control group and one from ILC2 *Lcr1* ^−/−^ group. Error bars = SEM.

**Figure S2.** Lung innate immune cells at day 22 post PR8‐infection. (a) Changes in body weight throughout the course of infection in *Pou2f3* ^+/−^ (*n* = 5) and *Pou2f3* ^−/−^ (*n* = 6) mice. (b) Total lung cell numbers of *Pou2f3* ^+/−^ and *Pou2f3* ^−/−^ mice at 22 days (D22) following PR8‐infection (p.i.). (c) Frequency of CD127^+^ and total cell numbers of ILC1s (Rorgt^−^Tbet^+^ of KLRG1^−^CD127^+^cells) at D22 p.i. in *Pou2f3* ^+/−^ and *Pou2f3* ^−/−^ mice. (d) Representative contour plots of lung ILC2s (KLRG1^+^NK1.1^−^ of CD127^+^ Lin^−^ cells) at D22 p.i. in *Pou2f3* ^+/−^ and *Pou2f3* ^−/−^ mice. (e) Representative contour plots of lung eosinophils (Eos: CD11c^low/int^ MHCII^−^ of SiglecF^+^F4/80^+^ cells) and alveolar macrophages (Alv. Mac.: CD11c^high^ MHCII^+^ of SiglecF^+^F4/80^+^ cells) in the lungs of *Pou2f3* ^+/−^ and *Pou2f3* ^−/−^ mice at D22 p.i. (f) Frequency of CD45^+^ and total cell numbers of alveolar macrophages (CD11c^high^ MHCII^+^ of SiglecF^+^F4/80^+^ cells), (g) inflammatory monocytes (Ly6c^high^CD11b^+^ of Ly6g^−^ cells) and (h) natural killer cells (CD127^−^NK1.1^+^ of Lin^−^CD11b^low/int^ cells) in the lungs of *Pou2f3* ^+/−^ and *Pou2f3* ^−/−^ mice at D22 p.i. (b and f‐h) represent combined two independent experiments. Each circle represents an individual mouse. *p* values were calculated using unpaired, two‐tailed parametric Welch's *t*‐test (NS = non‐significant). Error bars = SEM. Complete gating strategy for the different immune cell populations found in material and methods.

**Figure S3.** Transcriptional profiling of immune cell populations in tuft cell‐deficient mice post‐influenza. (a) Single‐cell RNA‐seq UMAP clustering of sorted immune (CD45^+^) cell types from mice lungs at D22 p.i. (b) Volcano plot comparing gene expression in uninfected *Pou2f3* ^+/−^ and *Pou2f3* ^−/−^ mice (c) Volcano plot comparing gene expression in infected *Pou2f3* ^+/−^ and *Pou2f3* ^−/−^ mice lungs at D22 p.i. Top 12 differentially expressed genes (DEGs) and selected marker genes in (d) lymphocytes and (e) myeloid cells in *Pou2f3* ^+/−^ and *Pou2f3* ^−/−^ mice lungs at D22 p.i.

**Figure S4.** Luminex® cytokine array of bronchoalveolar lavage fluid collected from *Pou2f3* ^−/−^ mice and controls at days 9 and 22 post–PR8 infection. Protein concentration of cytokines collected from the bronchoalveolar lavage fluid (BALF) of *Pou2f3* ^+/−^ and *Pou2f3* ^−/−^ mice at D9 and D22 p.i. (a–f) Protein concentrations of IL‐1α, IL‐1β, eotaxin, IL‐5, IL‐6, and LIF at D9 p.i. in the BALF of *Pou2f3* ^+/−^ (*n* = 6) and *Pou2f3* ^−/−^ (*n* = 5) mice. (g–m) Protein concentrations of IL‐1α, IL‐1β, eotaxin, IL‐5, CXCL5/LIX, IL‐6, and LIF at D22 p.i in the BALF of *Pou2f3* ^+/−^ (*n* = 8) and *Pou2f3* ^−/−^ (*n* = 9) mice. g‐m combined two independent experiments. Each circle represents an individual mouse. Statistical testing utilized a value of zero when protein concentration was below the limit of detection. Statistical significance was determined using the nonparametric Mann–Whitney test because of the small sample size and non‐normal distribution of the data. (NS = non‐significant). Error bars = SEM.

**Figure S5.** Changes in body weight and oxyhemoglobin saturations following PR8 infection and subsequent allergen challenge. (a) Body weight was measured at indicated time points following PR8 infection and *Alternaria alternata* challenge. Mice were initially infected with PR8 and allowed to recover, then sensitized on D22 with *A. alternata* (40 μg) or vehicle control (PBS), followed by challenge on D29, D30, D31, D33, D34, and D35 with *A. alternata* (20 μg) or PBS and harvested at D38 post‐PR8. (b) Total lung cell numbers in *Pou2f3* ^+/−^ and *Pou2f3* ^−/−^ mice at D38 p.i. following challenge with PBS or *A. alternata*; *n* = 4/group. (c) Pulse oximetry were assessed at indicated time points following PR8 infection challenge with PBS or *A. alternata*. (a, c) *Pou2f3* ^*+/−*^ *PBS (n = 4), Pou2f3* ^*−/−*^ *PBS (n = 4), Pou2f3* ^*+/−*^ *A. alternata (n = 7), and Pou2f3* ^*−/−*^ *A. alternata (n = 6). p* values were calculated using one‐way ANOVA with Tukey's post test for multiple comparisons for each time point collected (NS = non‐significant). Error bars = SEM.

**Figure S6.** Lung innate immune cells following PR8 infection and subsequent *Alternaria alternata* challenge. (a) Frequency of CD127^+^ and total cell numbers of ILC1s (NK1.1^+^KLRG1^−^ of CD127^+^Lin^−^ cells) in *Pou2f3* ^+/−^ and *Pou2f3* ^−/−^ mice at D38 p.i. following challenge with PBS or *A. alternata*. (b) Total numbers of alveolar macrophages (CD11c^+^MHCII^+^ of SiglecF^+^CD11B^int/high^ cells), (c) inflammatory monocytes (Ly6c^high^CD11b^+^ of Ly6g^−^ cells) and (d) natural killer cells (NK1.1^+^ CD127^−^ of Lin^−^ cells) in *Pou2f3* ^+/−^ and *Pou2f3* ^−/−^ mice at D38 p.i. following challenge with PBS or *A. alternata*. Each circle represents an individual mouse. *p* values were calculated using one‐way ANOVA with Tukey's post test for multiple comparisons. (NS = non‐significant). Error bars = SEM. Complete gating strategy for the different immune cell populations found in material and methods.

## Data Availability

Single cell sequencing data can be accessed using the GEO accession numbers listed below. RNA‐seq profiling of uninfected and infected *Pou2f3* ^+/−^ and *Pou2f3* ^−/−^ mice at D22 following infection: *non‐immune cells* (CD45^−^) only; GSE320109. RNA‐seq profiling of uninfected and infected *Pou2f3* ^+/−^ and *Pou2f3* ^−/−^ mice at D22 following infection: *immune cells* (CD45^+^) only; GSE320111.
