# Supplementary figures and images for: Influenza‐induced tuft cell expansion is associated with changes in ILC2 populations in the distal lungs of mice
Source: Physiol Rep. 2026 Jul 11;14(13):e71000. doi: 10.14814/phy2.71000 (PMC13356277; doi:10.14814/phy2.71000)

Supplemental Figure 1

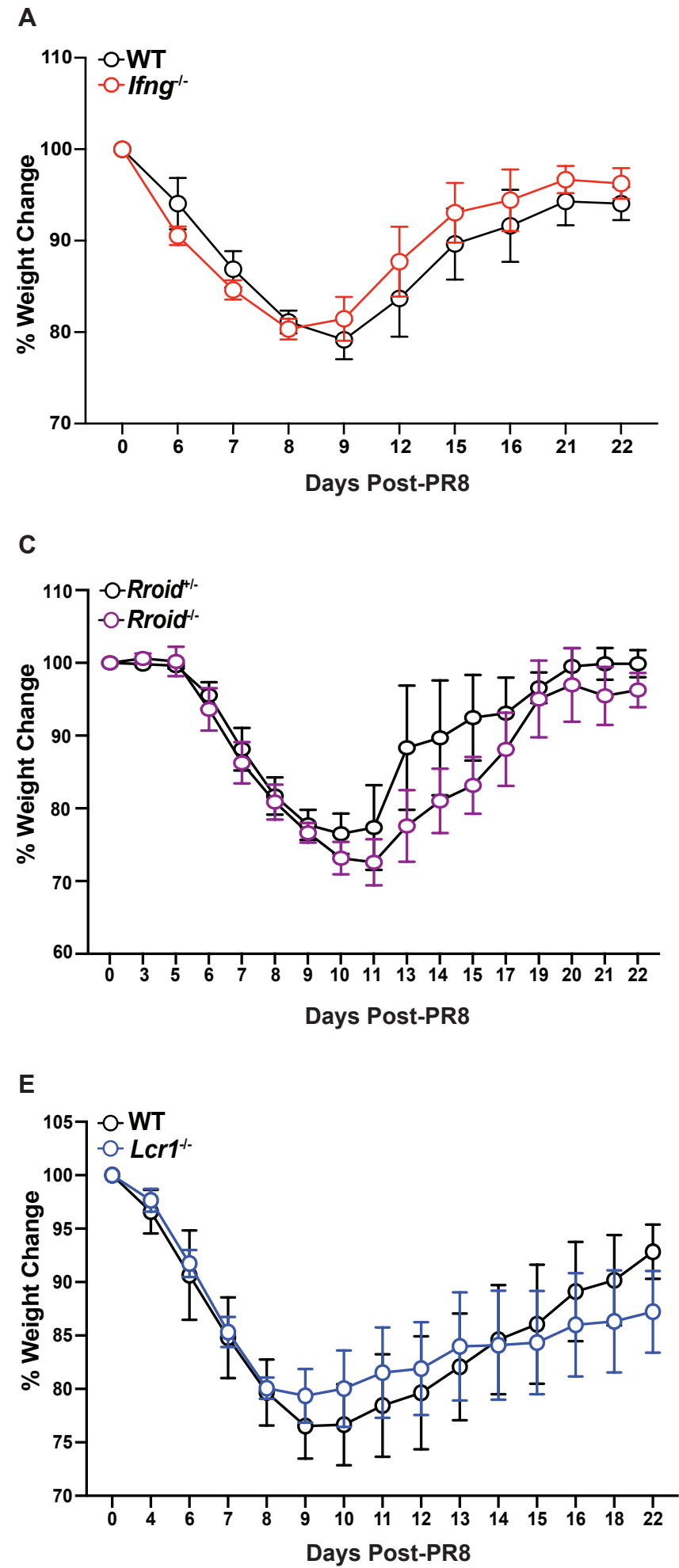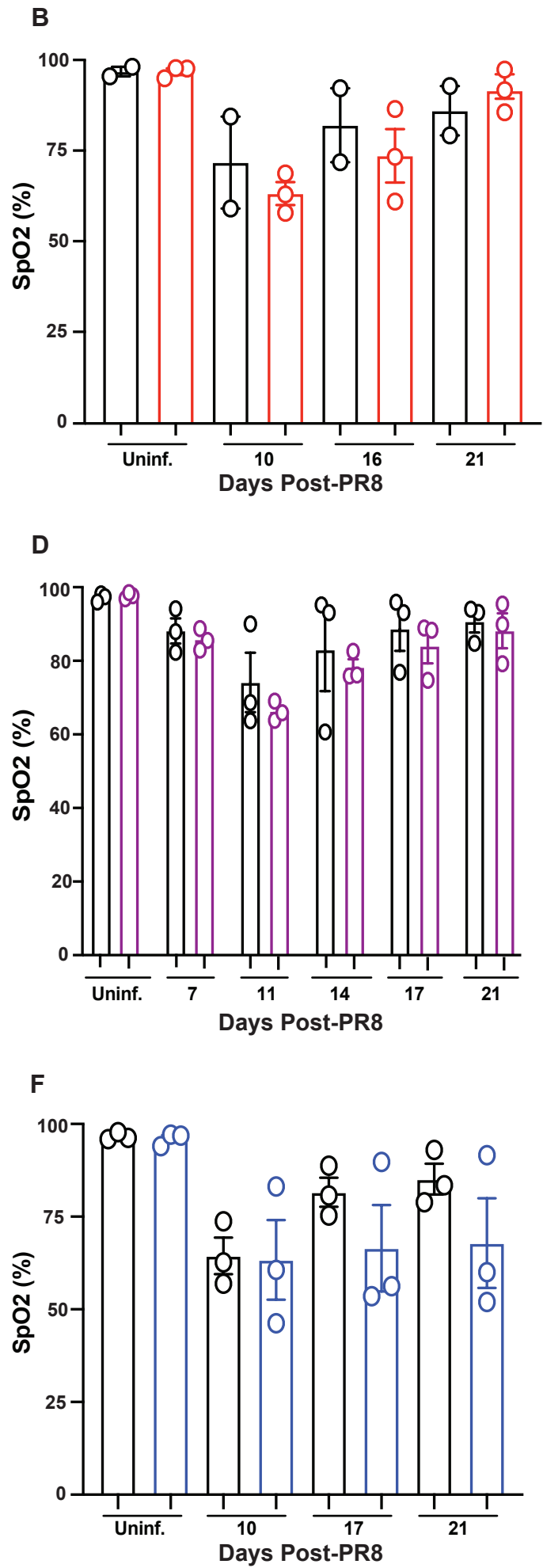

Supplement: Supplementary file 1 — Figure S1. Changes in body weight and oxyhemoglobin saturations following PR8 infection. Body weight and pulse oximetry were assessed prior to infection and at indicated time points following PR8 infection. (A, B) WT C57BL/6J (n = 2–5) and Ifng −/− (n = 3–8) mice, (C, D) Rroid +/− (n = 3), and Rroid −/− (n = 3) mice and (E, F) Lcr1 ‐/− (n = 3–5) and WT C57BL/6J (n = 3–5) mice. (A, E) combined two independent experiments. (B, D and F) each circle represents an individual mouse. (E) Two mice that lost <15% of starting body weight after influenza infection were excluded, one from control group and one from ILC2 Lcr1 −/− group. Error bars = SEM. [file PHY2-14-e71000-s004.pdf]

Supplemental Figure 2

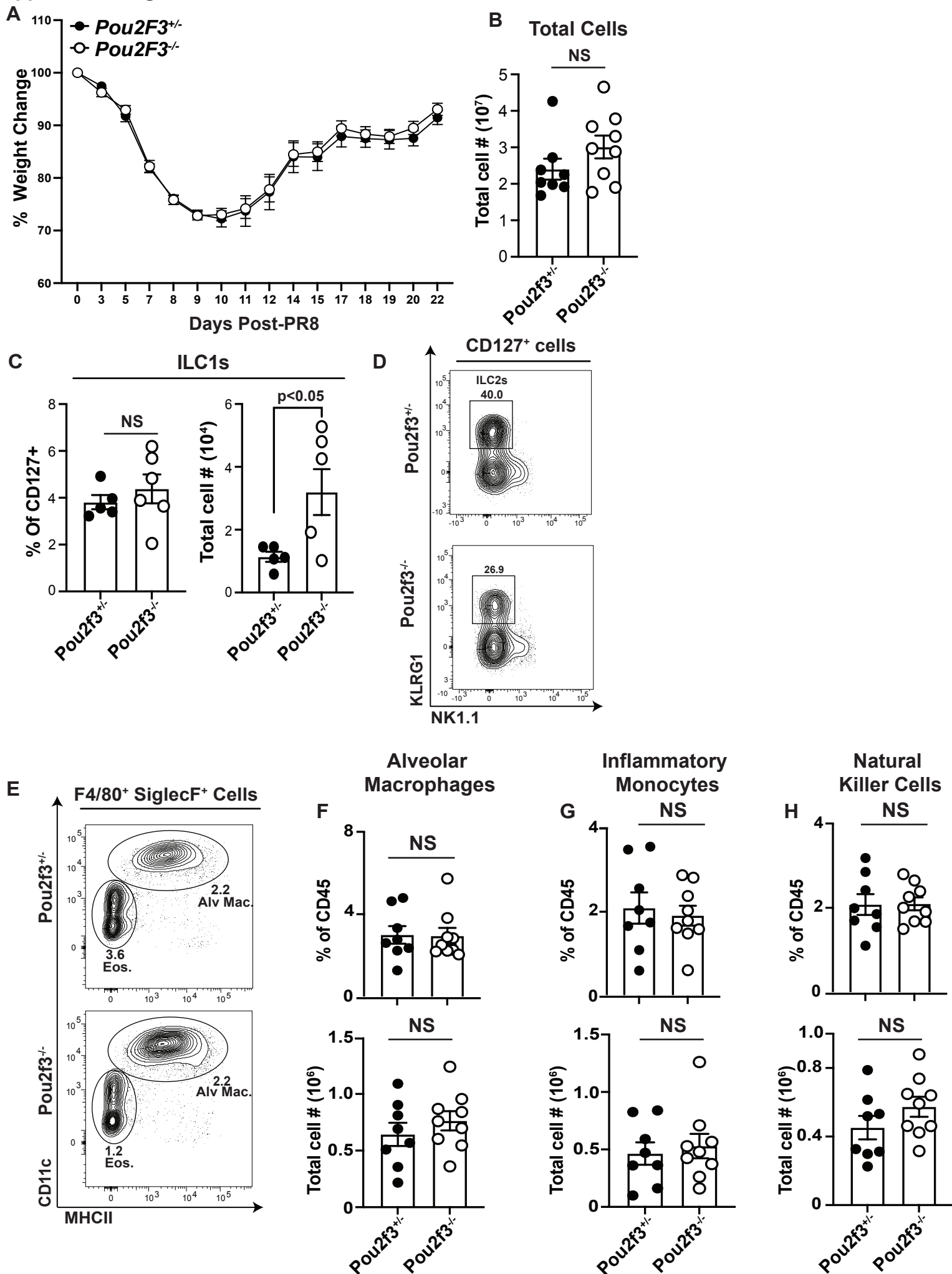

Supplement: Supplementary file 2 — Figure S2. Lung innate immune cells at day 22 post PR8‐infection. (a) Changes in body weight throughout the course of infection in Pou2f3 +/− (n = 5) and Pou2f3 −/− (n = 6) mice. (b) Total lung cell numbers of Pou2f3 +/− and Pou2f3 −/− mice at 22 days (D22) following PR8‐infection (p.i.). (c) Frequency of CD127+ and total cell numbers of ILC1s (Rorgt−Tbet+ of KLRG1−CD127+cells) at D22 p.i. in Pou2f3 +/− and Pou2f3 −/− mice. (d) Representative contour plots of lung ILC2s (KLRG1+NK1.1− of CD127+ Lin− cells) at D22 p.i. in Pou2f3 +/− and Pou2f3 −/− mice. (e) Representative contour plots of lung eosinophils (Eos: CD11clow/int MHCII− of SiglecF+F4/80+ cells) and alveolar macrophages (Alv. Mac.: CD11chigh MHCII+ of SiglecF+F4/80+ cells) in the lungs of Pou2f3 +/− and Pou2f3 −/− mice at D22 p.i. (f) Frequency of CD45+ and total cell numbers of alveolar macrophages (CD11chigh MHCII+ of SiglecF+F4/80+ cells), (g) inflammatory monocytes (Ly6chighCD11b+ of Ly6g− cells) and (h) natural killer cells (CD127−NK1.1+ of Lin−CD11blow/int cells) in the lungs of Pou2f3 +/− and Pou2f3 −/− mice at D22 p.i. (b and f‐h) represent combined two independent experiments. Each circle represents an individual mouse. p values were calculated using unpaired, two‐tailed parametric Welch's t‐test (NS = non‐significant). Error bars = SEM. Complete gating strategy for the different immune cell populations found in material and methods. [file PHY2-14-e71000-s003.pdf]

Supplemental Figure 3

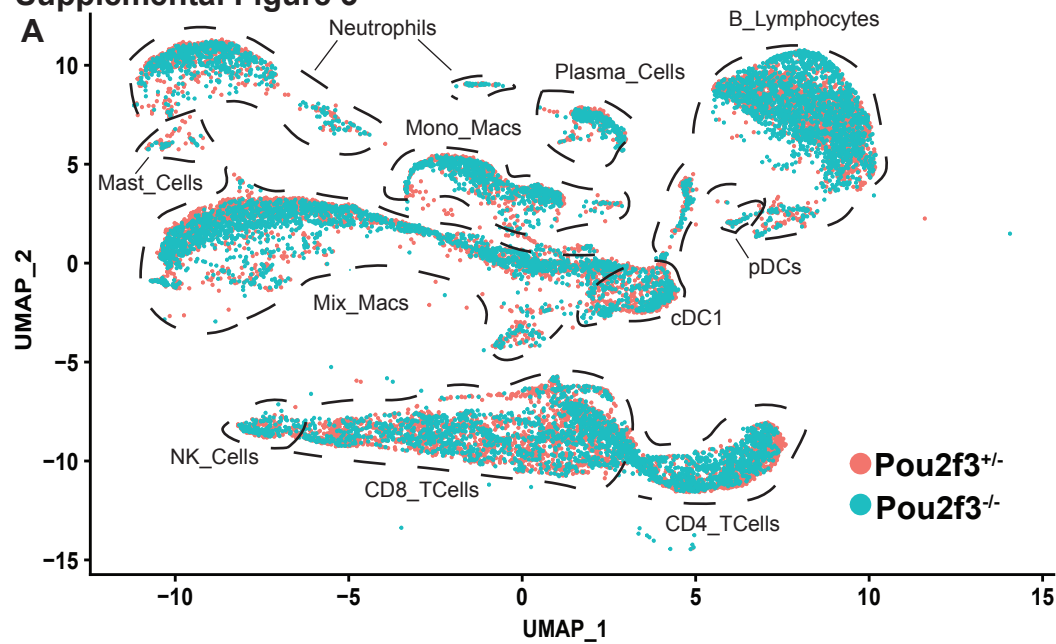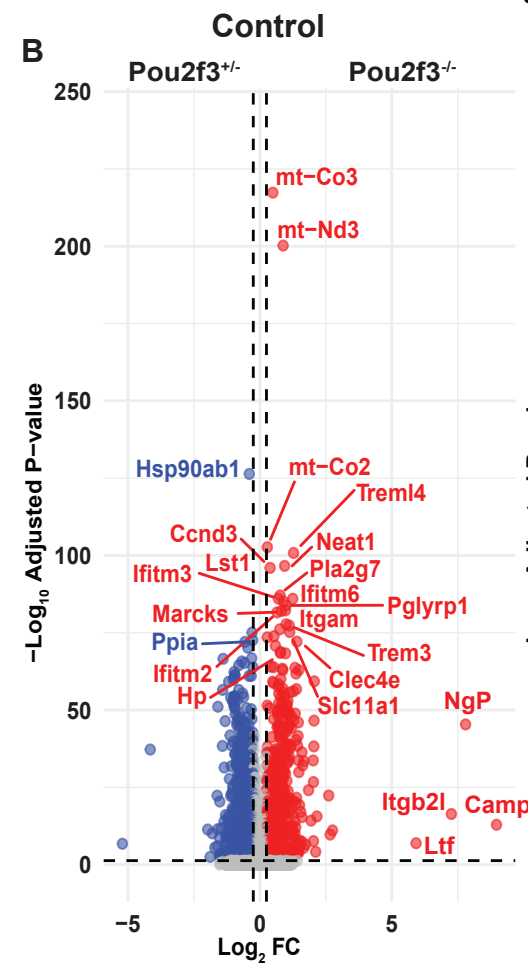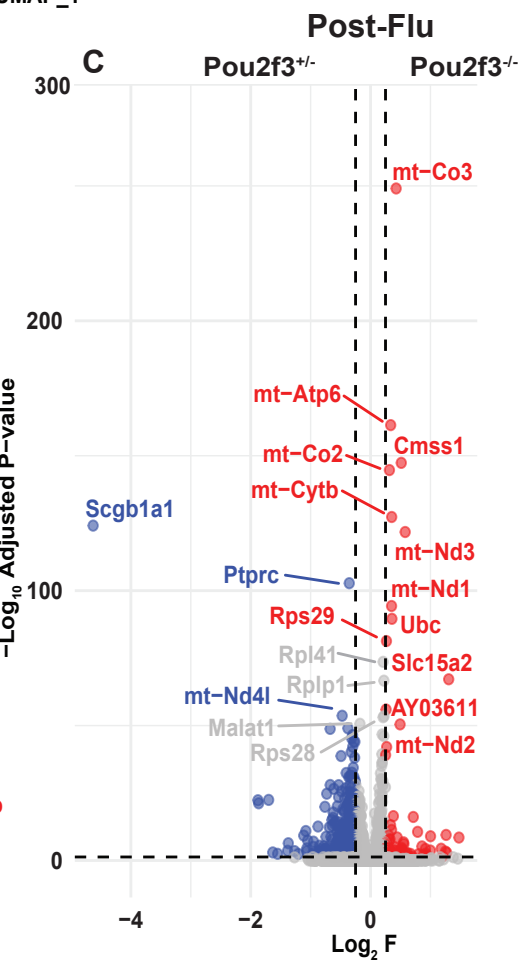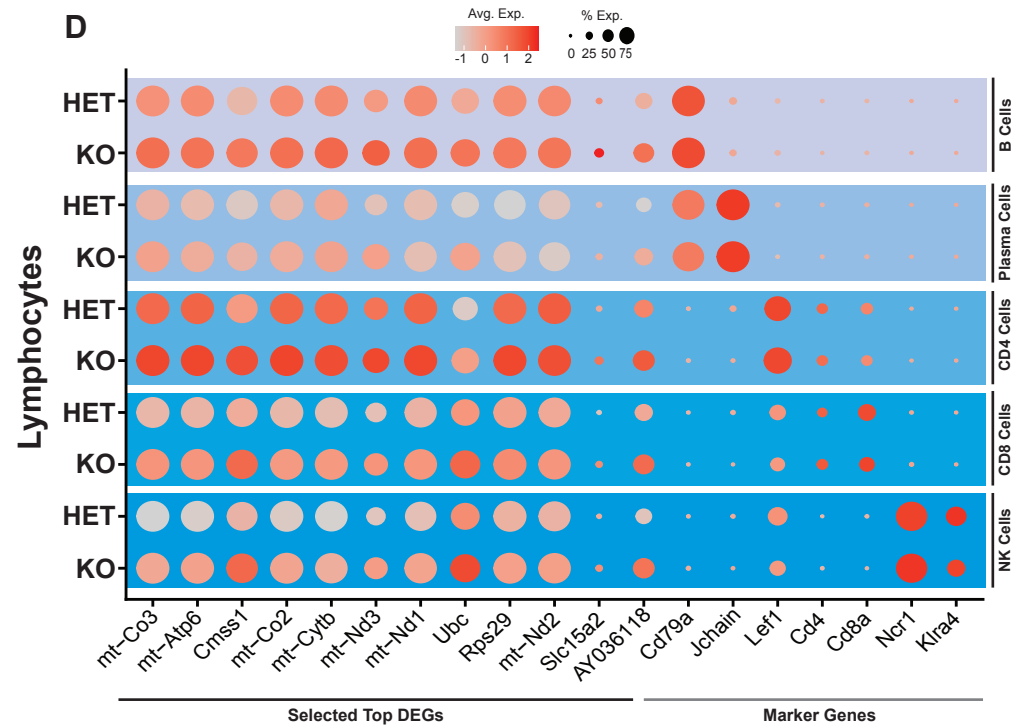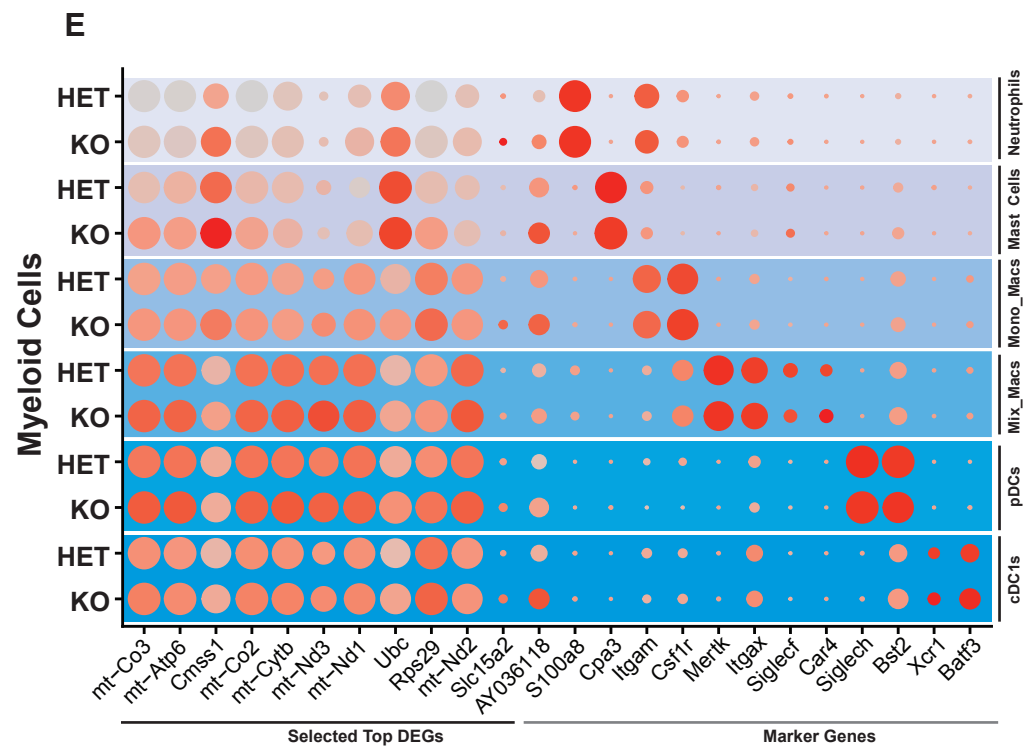

Supplement: Supplementary file 3 — Figure S3. Transcriptional profiling of immune cell populations in tuft cell‐deficient mice post‐influenza. (a) Single‐cell RNA‐seq UMAP clustering of sorted immune (CD45+) cell types from mice lungs at D22 p.i. (b) Volcano plot comparing gene expression in uninfected Pou2f3 +/− and Pou2f3 −/− mice (c) Volcano plot comparing gene expression in infected Pou2f3 +/− and Pou2f3 −/− mice lungs at D22 p.i. Top 12 differentially expressed genes (DEGs) and selected marker genes in (d) lymphocytes and (e) myeloid cells in Pou2f3 +/− and Pou2f3 −/− mice lungs at D22 p.i. [file PHY2-14-e71000-s006.pdf]

Supplemental Figure 5

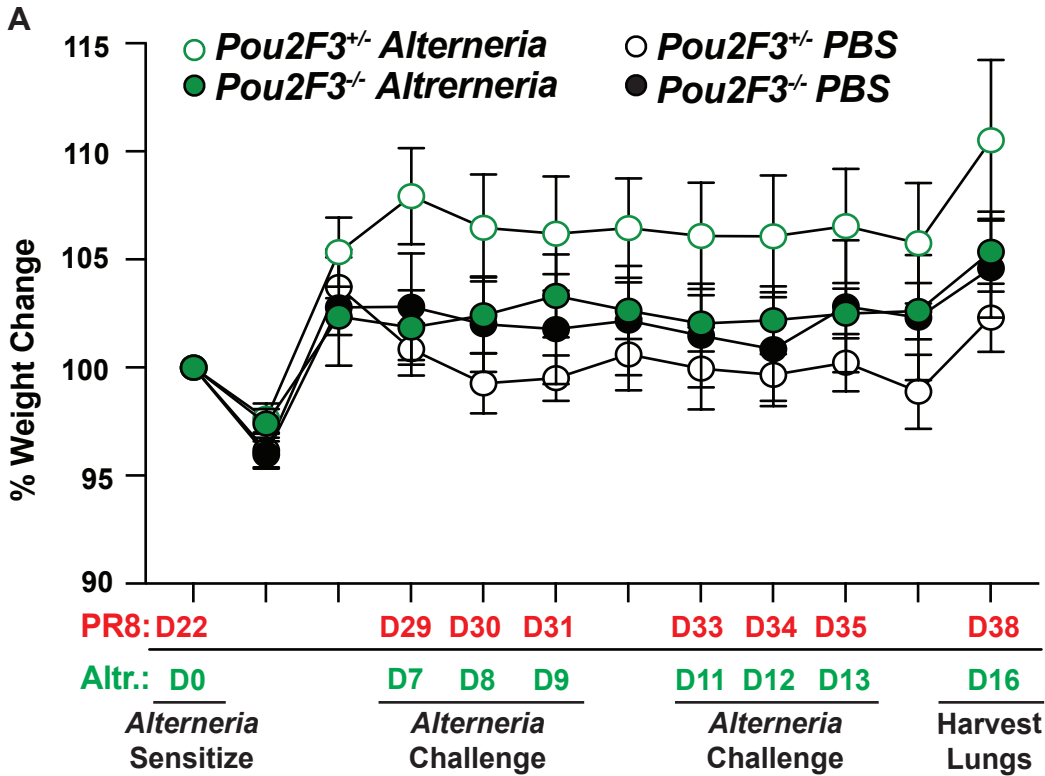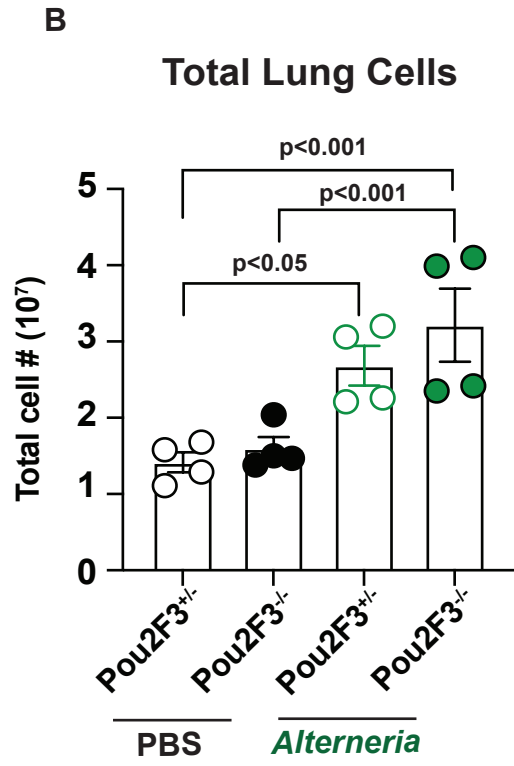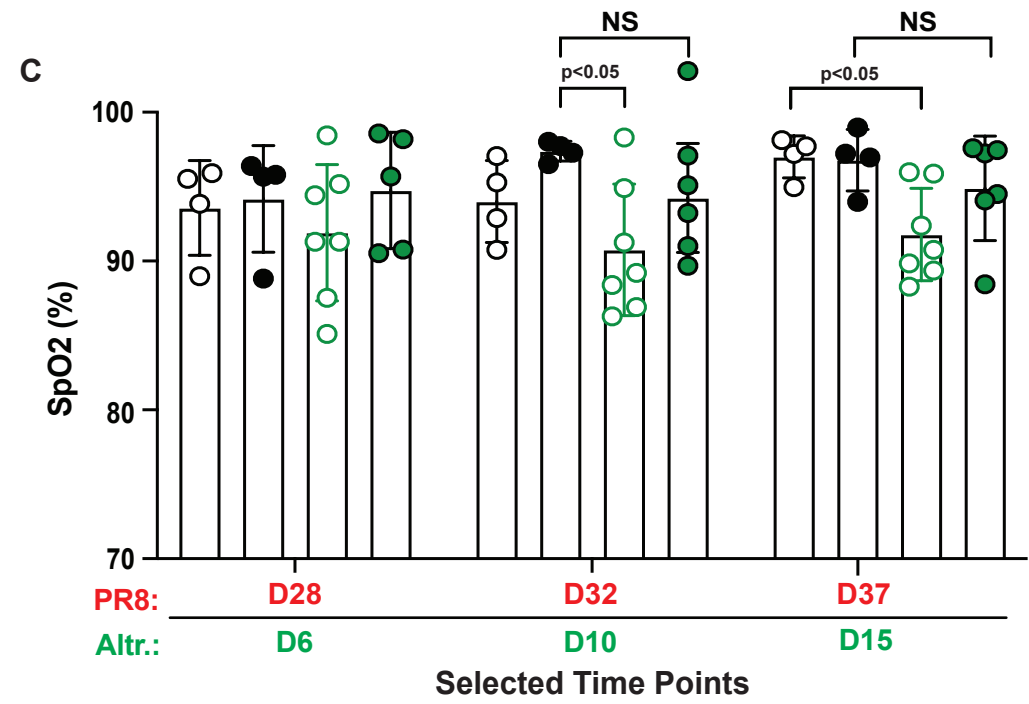

Supplement: Supplementary file 5 — Figure S5. Changes in body weight and oxyhemoglobin saturations following PR8 infection and subsequent allergen challenge. (a) Body weight was measured at indicated time points following PR8 infection and Alternaria alternata challenge. Mice were initially infected with PR8 and allowed to recover, then sensitized on D22 with A. alternata (40 μg) or vehicle control (PBS), followed by challenge on D29, D30, D31, D33, D34, and D35 with A. alternata (20 μg) or PBS and harvested at D38 post‐PR8. (b) Total lung cell numbers in Pou2f3 +/− and Pou2f3 −/− mice at D38 p.i. following challenge with PBS or A. alternata; n = 4/group. (c) Pulse oximetry were assessed at indicated time points following PR8 infection challenge with PBS or A. alternata. (a, c) Pou2f3 +/− PBS (n = 4), Pou2f3 −/− PBS (n = 4), Pou2f3 +/− A. alternata (n = 7), and Pou2f3 −/− A. alternata (n = 6). p values were calculated using one‐way ANOVA with Tukey's post test for multiple comparisons for each time point collected (NS = non‐significant). Error bars = SEM. [file PHY2-14-e71000-s001.pdf]

Supplemental Figure 6

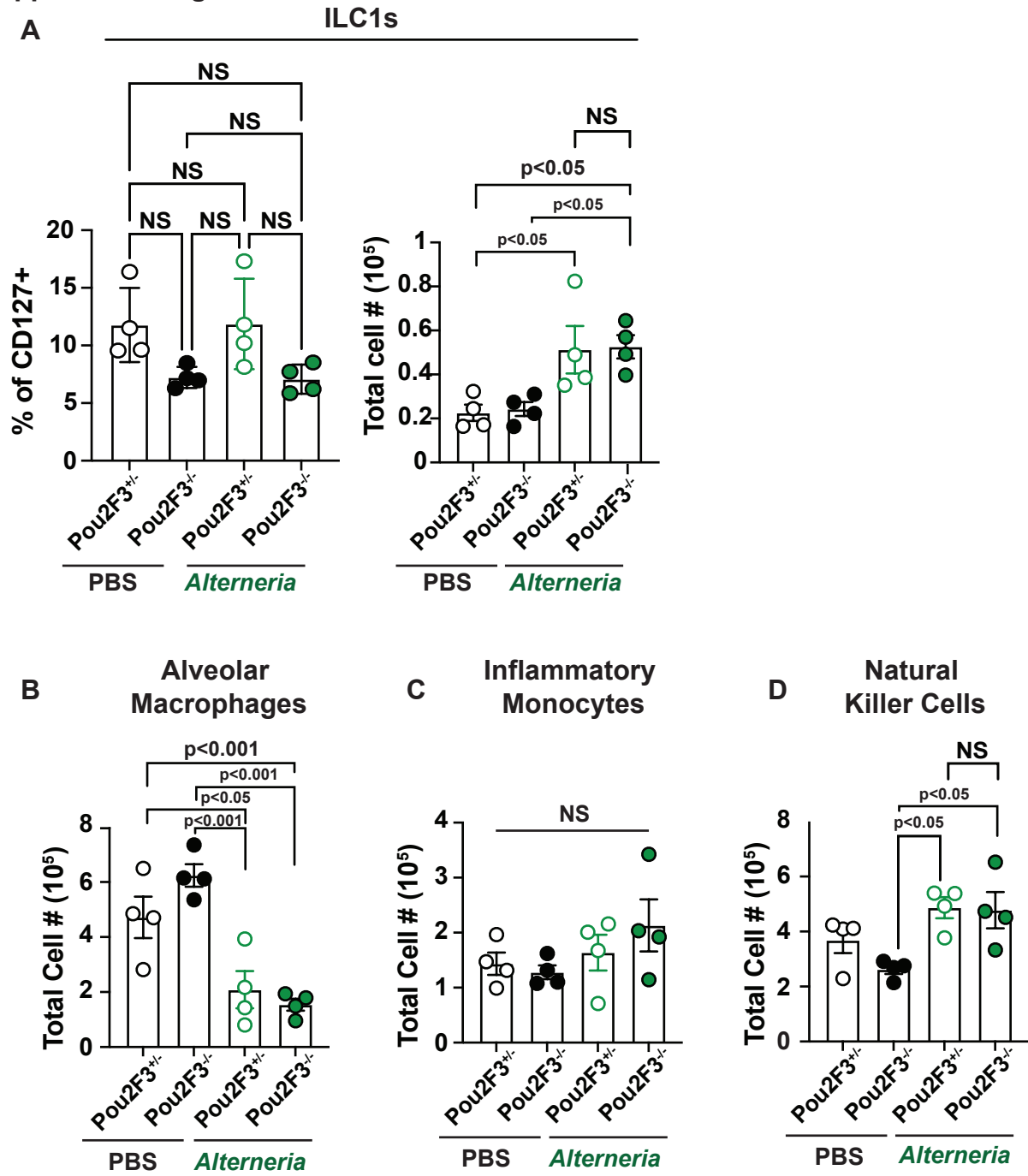

Supplement: Supplementary file 6 — Figure S6. Lung innate immune cells following PR8 infection and subsequent Alternaria alternata challenge. (a) Frequency of CD127+ and total cell numbers of ILC1s (NK1.1+KLRG1− of CD127+Lin− cells) in Pou2f3 +/− and Pou2f3 −/− mice at D38 p.i. following challenge with PBS or A. alternata. (b) Total numbers of alveolar macrophages (CD11c+MHCII+ of SiglecF+CD11Bint/high cells), (c) inflammatory monocytes (Ly6chighCD11b+ of Ly6g− cells) and (d) natural killer cells (NK1.1+ CD127− of Lin− cells) in Pou2f3 +/− and Pou2f3 −/− mice at D38 p.i. following challenge with PBS or A. alternata. Each circle represents an individual mouse. p values were calculated using one‐way ANOVA with Tukey's post test for multiple comparisons. (NS = non‐significant). Error bars = SEM. Complete gating strategy for the different immune cell populations found in material and methods. [file PHY2-14-e71000-s002.pdf]
