# Supplementary material for: Influenza‐induced tuft cell expansion is associated with changes in ILC2 populations in the distal lungs of mice
Source: Physiol Rep. 2026 Jul 11;14(13):e71000. doi: 10.14814/phy2.71000 (PMC13356277; doi:10.14814/phy2.71000)

Supplemental Figure 4

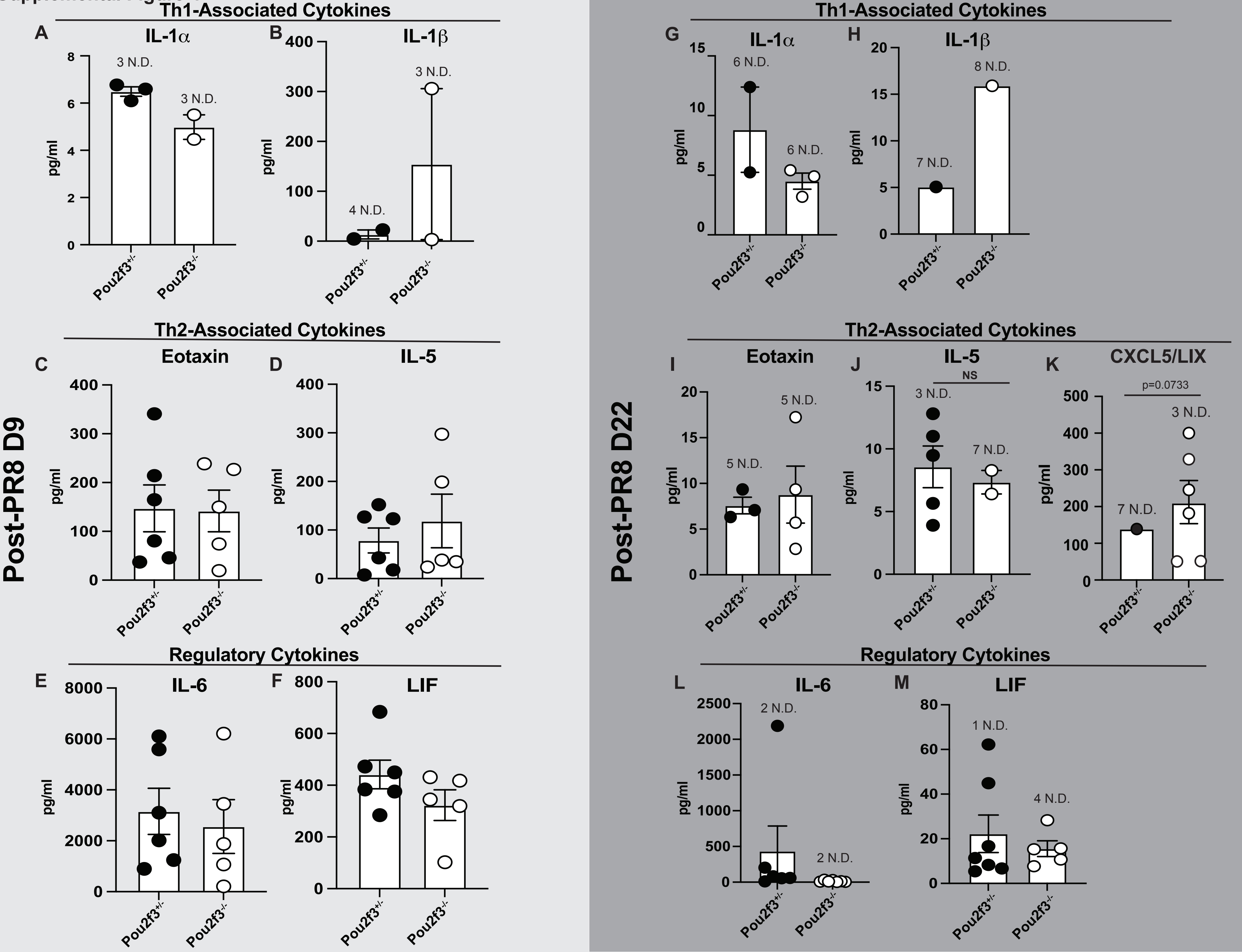

Supplement: Supplementary file 4 — Figure S4. Luminex® cytokine array of bronchoalveolar lavage fluid collected from Pou2f3 −/− mice and controls at days 9 and 22 post–PR8 infection. Protein concentration of cytokines collected from the bronchoalveolar lavage fluid (BALF) of Pou2f3 +/− and Pou2f3 −/− mice at D9 and D22 p.i. (a–f) Protein concentrations of IL‐1α, IL‐1β, eotaxin, IL‐5, IL‐6, and LIF at D9 p.i. in the BALF of Pou2f3 +/− (n = 6) and Pou2f3 −/− (n = 5) mice. (g–m) Protein concentrations of IL‐1α, IL‐1β, eotaxin, IL‐5, CXCL5/LIX, IL‐6, and LIF at D22 p.i in the BALF of Pou2f3 +/− (n = 8) and Pou2f3 −/− (n = 9) mice. g‐m combined two independent experiments. Each circle represents an individual mouse. Statistical testing utilized a value of zero when protein concentration was below the limit of detection. Statistical significance was determined using the nonparametric Mann–Whitney test because of the small sample size and non‐normal distribution of the data. (NS = non‐significant). Error bars = SEM. [file PHY2-14-e71000-s005.pdf]
